# Characteristics of Harmful Algal Species in the Coastal Waters of China from 1990 to 2017

**DOI:** 10.3390/toxins14030160

**Published:** 2022-02-23

**Authors:** Wanli Hou, Xi Chen, Menglin Ba, Jianghua Yu, Tiantian Chen, Yihui Zhu, Jie Bai

**Affiliations:** 1 Key Laboratory of Marine Environmental Science and Ecology of Ministry of Education, College of Environmental Science and Engineering, Ocean University of China, Qingdao 266100, China; houwanli@stu.ouc.edu.cn (W.H.); baijie@ouc.edu.cn (J.B.); 2 Marine Ecology Laboratory, College of Marine Life Sciences, Ocean University of China, Qingdao 266003, China; chenxi@ouc.edu.cn; 3 Jiangsu Key Laboratory of Atmospheric Environment Monitoring and Pollution Control, School of Environmental Science and Engineering, Nanjing University of Information Science & Technology, Nanjing 210044, China; bamenglin666@163.com (M.B.); zhuyihui2536@163.com (Y.Z.); 4 CAS Key Laboratory of Marine Ecology and Environmental Sciences, Institute of Oceanology, Chinese Academy of Sciences, Qingdao 266071, China; chentian0819@163.com

**Keywords:** harmful algal blooms, cyst, long-term, Chinese coastal waters

## Abstract

Harmful algal blooms (HABs) have occurred frequently in coastal waters of China, imposing negative effects on the marine ecological environment. A dataset of HABs and terrestrial runoff was collected and analyzed in this study, and factors responsible for HABs were further explored. Frequency and expansion of HABs peaked between 2001 and 2007, and although they have declined slightly since then, they have remained quite high. Frequency and accumulative area of HABs peaked in 2004–2005, and most occurred from April to August during these years. HABs occurred frequently in the Changjiang (Yangtze River) estuary, and *Prorocentrum donghaiense*, *Noctiluca scientillans*, *Karenia mikimotoi,* and *Skeletonema costatum* were the main algal species. The increases of eutrophication, the abnormal sea surface temperature caused by climate and ocean currents, and the species invasion caused by the discharge of ballast water may be important factors for the long-term outbreak of HABs in the Chinese coastal waters. These findings provide a better understanding of HABs in China, which will be helpful to further prevention and control.

## 1. Introduction

Harmful algal blooms (HABs) are the kind of ecological anomaly, which are accompanied by the rapid proliferation and aggregation of microplankton [1,2,3]. HABs change the structure and function of freshwater and marine ecosystems, destroy fisheries resources and aquatic environments [4,5], and result in toxic seafood and toxicity to human health [6,7,8]. With the rapid economic development and urbanization in Chinese coastal areas, marine ecological environment pollution and eutrophication have become increasingly serious in recent years, HABs have been recorded in many coastal regions, and algal toxins have been found in most of them, resulting in deleterious impacts [9,10,11]. It is vital to assess the HABs trends in China due to the growing effects of algal toxins on marine ecosystem and human health.

Chinese marine environment is very diverse, with the sea areas spanning the warm temperate, subtropical, and tropical climate zones, and there is not only inland sea with poor water exchange capacity, but also the edge of the sea has an active ocean current, which facilitates HABs. Much effort has been expended to investigate the mechanism(s) of abiotic factors in regulating HABs, mainly referring to environmental parameters and nutritional conditions [12,13,14,15]. The intensity and frequency of HABs have increased in the past few decades, which has attracted attention to the evolution of HABs. Therefore, scholars used the regionally integrated approach to study the long-term HABs in some areas [16,17,18] and even the whole Chinese coastal area [19,20].

Although some articles reported the long-term regularities and species changes of HABs in China [16,17,18,19,20], the causes and effects are not fully understood. Therefore, this paper analyzes the long-term (1990–2017) data of HABs in Chinese coastal waters, summarizes the characteristics, and explores the main influencing factors of HAB-related species so as to provide scientific theory and basis for Chinese targeted prevention and control of HABs.

## 2. Results

### 2.1. Occurrence of HABs in China

**Figure 1:**
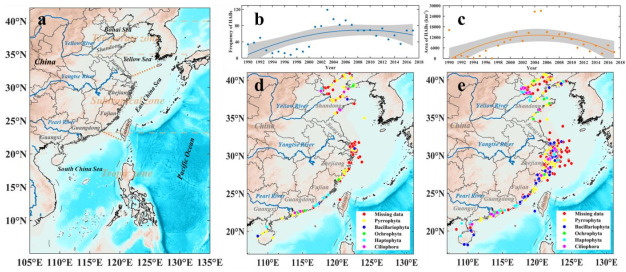
The coastal waters of China (**a**), frequency (**b**), and area (**c**) of HABs in China from 1990 to 2017 and locations of HABs in 1990–2000 (**d**) and after 2000 (**e**). Note: different color spots represent different algal species at phylum level.

A total of 1489 HABs were recorded in the Chinese coastal waters from 1990 to 2017, and the total area affected by HABs amounted to 250,392 km^2^ (Table S1). The distribution of HABs in China exhibited a clear time-dependent trend: the frequency and area of HABs increased before 2003 and then declined (Figure 1b,c). The annual frequency reached 53 events, with the annual affected areas of 8943 km^2^ during the period from 1990 to 2017. The frequency and area of HABs in 1990–1999 were relatively low, with an average of 23 events and 4180 km^2^ per year. From 2000 to 2010, the annual frequency and affected area of HABs were 78 events and 15,383 km^2^, respectively, which were 3.39 and 3.68 times that of 1990–1999. From 2011 to 2017, the annual frequency and area of HABs were 57 and 5626 km^2^, showing a 2.49-fold and 1.35-fold increase compared with that of 1990 to 1999.

The event-prone area of HABs are mainly located in Bohai Bay, the Yangtze River Estuary, and the Pearl River Estuary (Table S2). From 1990 to 2000, HABs mainly broke out in the Bohai Sea area, the Pearl River Estuary, and the Fujian Zhejiang coast of the East China Sea (Figure 1d). After 2000, HABs were recorded in the whole coastline of the Bohai Sea and the East China Sea. HABs mainly occurred in the north of the Yellow Sea and also occurred in the South China Sea, mainly occurring in the waters near the Pearl River Estuary (Figure 1e). In addition, it can be seen that the HABs extended to outer sea and appeared in the area around 200 miles away from the shore in 2017, which was only 50 miles in 1990; among them, the main related species of HABs are dinoflagellates and diatoms.

### 2.2. Interannual Variation of HABs

**Figure 2:**
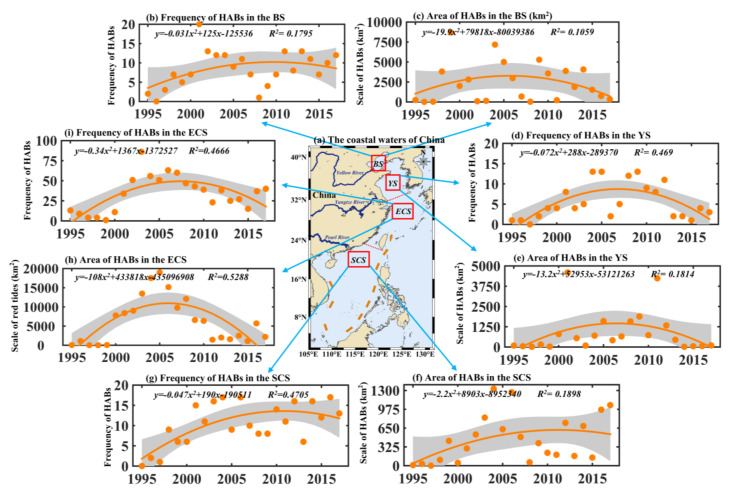
(**a**) The coastal waters of China. Long-term changes of HABs frequency (**b**) and area (**c**) in the Bohai Sea (BS). Long-term changes of HABs frequency (**d**) and area (**e**) in the Yellow Sea (YS). Long-term changes of HABs area (**f**) and frequency (**g**) in the South China Sea (SCS). Long-term changes of HABs area (**h**) and frequency (**i**) in the East China Sea (ECS).

As shown in Figure 2, the trend of HABs in each sea was to increase first and then decrease. However, the frequency and accumulative area differed in the four sea areas. There were more than 33 annual HABs in the East China Sea from 1995 to 2017, with an average annual cumulative area of 6215 km^2^, and in terms of quantity and area, this far exceeded other sea areas. The HABs occurred in the East China Sea more than 50 annual times from 2003 to 2007, with an average annual cumulative area of about 14,500 km^2^. The HABs occurred most frequently in 2003, and the maximum accumulative area occurred in 2005 (Figure 2h,i). The characteristics of HABs run a wide range in the Bohai Sea, and the average annual occurrence was eight times from 1995 to 2017, but the average annual accumulative area was 2398 km^2^, of which four HABs in 2009 caused 5279 km^2^ of sea area to be affected. and 12 HABs in 2004 caused 7180 km^2^ of sea area to be affected (Figure 2b,c). The frequency and area of HABs in the South China Sea and the Yellow Sea were relatively low, with the average annual frequency being 10 times and 5 times lower, and the average annual cumulative area being 462 km^2^ and 877 km^2^, respectively (Figure 2d–g, Table S3). 

### 2.3. Seasonal Pattern of HABs

**Figure 3:**
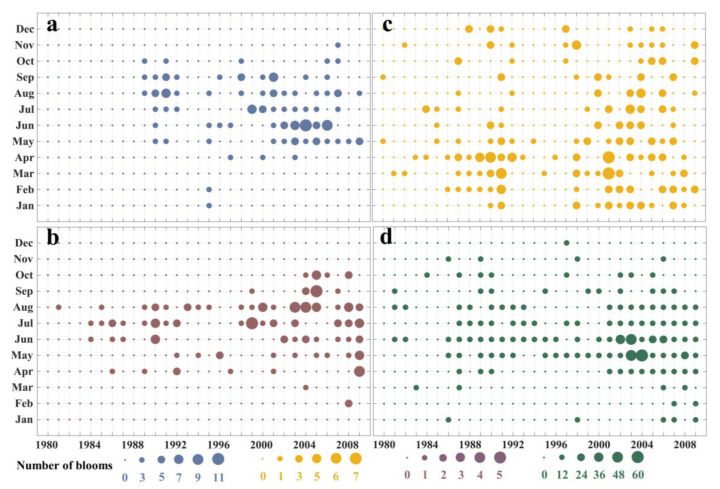
Monthly changes of HABs in the Bohai Sea (**a**), Yellow Sea (**b**), South China Sea (**c**), and East China Sea (**d**) during 1980 to 2009.

The seasonal pattern of HABs exhibited a different trend in each sea from 1980 to 2009 (Table S2), as illustrated in Figure 3. In the Bohai Sea, HABs occurred from May to September (Figure 3a), which accounted for 91% of the total. In the Yellow Sea, HABs occurred from June to August (Figure 3b). With increasing years, the first HABs occurred earlier in the year and ended later; hence, HABs have been lasting longer. In the South China Sea, the seasonal pattern variation of HABs was not obvious. HABs occurred every month, while most of the HABs were recorded from February to May (Figure 3c). In the East China Sea, HABs were mainly recorded from May to August (Figure 3d). Only 12 times were HABs recorded in November, December, January, and February during these years.

### 2.4. Related Species of HABs

**Figure 4:**
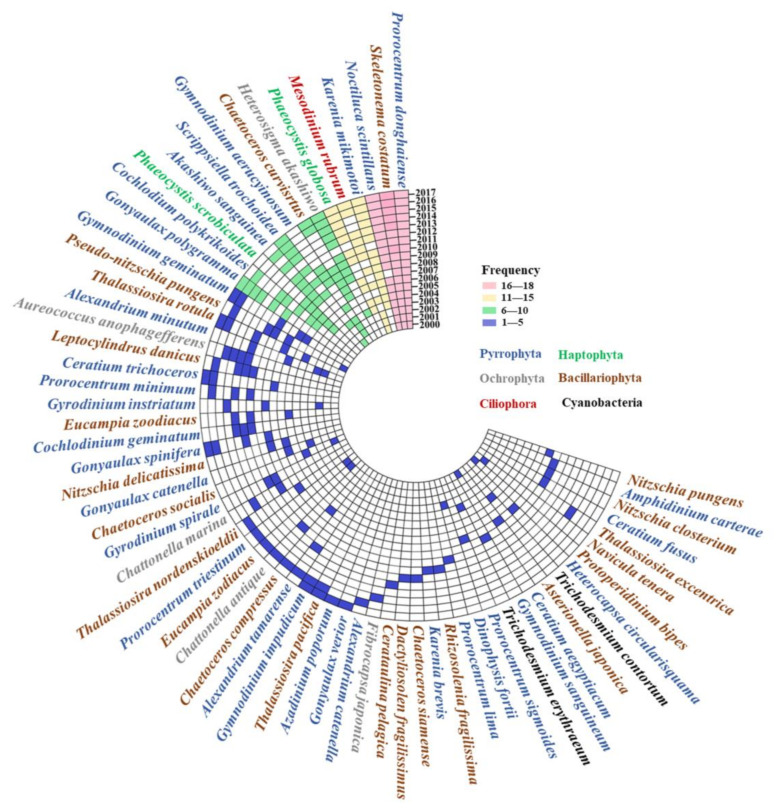
Frequency of dominant related species in the coastal waters of China from 2000 to 2017. Note: different colored squares represent different frequencies. The color of the words represents the algal species at the phylum level.

The total number of HAB-related species increased from 2000 to 2017 (Figure 4). Pyrrophyta is the main phylum of the algal species, followed by Bacillariophyta and Ochrophyta. Before 2010, *Prorocentrum donghaiense*, *Noctiluca,* and *Skeletonema costatum* were the dominant species responsible for HABs, which almost broke out every year, and *Heterosigma akashiwo*, *Karenia mikimotoi*, and *Phaeocystis globosa* were the increasingly dominant species responsible for HABs after 2010.

Sixty-four identified related species of HABs were collected in the Chinese coastal waters in the past decades. From 2000 to 2006, the dominant species were about nine each year and then increased sharply from 2008 to 2011 to about 18 species each year. The maximum number of dominant species was 28 in 2016.

### 2.5. Pollutants and Port Throughput

**Figure 5:**
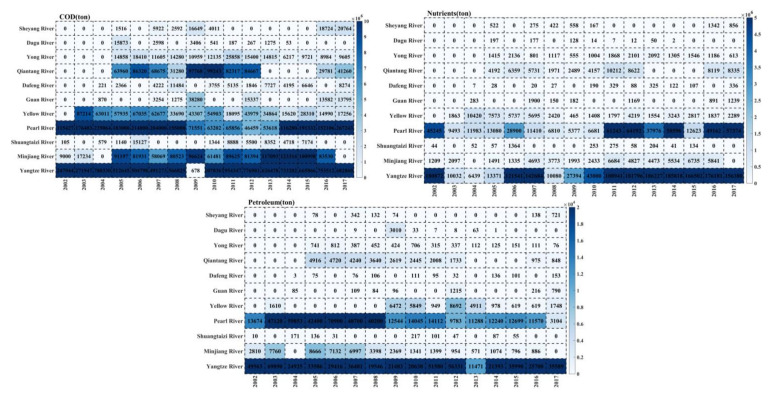
Changes of pollutants (nutrients/ton, COD/ton, petroleum/ton) in rivers entering the sea from 2002 to 2017.

The main rivers recorded in China had accumulated about 200 million tons of pollutants discharged into the ocean from 2002 to 2017 (Figure 5). Among them, COD, nutrients emissions, and petroleum pollutants accounted for 86%, 13%, and <1%, respectively. The Yangtze River, Pearl River, and Yellow River, respectively, discharged into the East China Sea, South China Sea, and Bohai Sea and accounted for 59.85%, 14.82%, and 3.66% of the total pollutant emissions, respectively.

**Figure 6:**
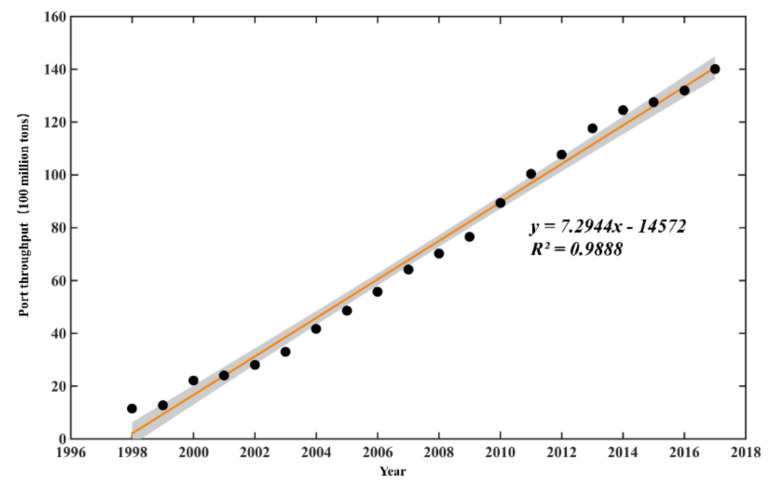
Changes of port throughput over years (1998–2017) in the coastal China Sea.

Port throughput is a quantitative reference for measuring the construction and development of countries, regions, and cities. Figure 6 shows the changes in the throughput of China’s coastal ports from 1998 to 2017. As can be seen from the figure, the throughput of China’s coastal ports increased year by year from 1.15 billion tons in 1998 to 14.007 billion tons in 2017.

### 2.6. Factors Driving the HABs

**Figure 7:**
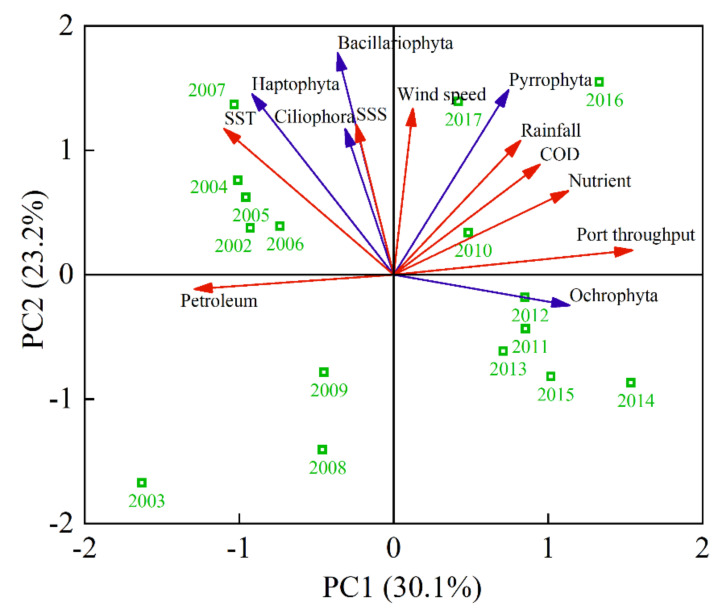
Principal components analysis of HAB-related species, causative species, and environmental variables in coastal China sea from 2002 to 2017. Pyrrophyta, Bacillariophyta, Haptophyta, Ochrophyta, and Ciliophora are causative groups of HABs. Environmental variables include rainfall, wind speed, port throughput, COD emissions, petroleum emissions, nutrient emissions, sea surface temperature (SST), and salinity (SSS).

The relationship between HAB-related species and environmental variables in the coastal China sea was analyzed using principal components analysis (Figure 7). Analysis of the two axes explained 30% and 23% of the total variance in the species environment relationship, respectively. Changes in Pyrrophyta were positively correlated with environmental variables, such as SSS, wind speed, rainfall, COD, and nutrients. Haptophyta, Ciliophora, and Bacillariophyta were closely correlated with SST, SSS, wind speed, and river runoff. Changes in Ochrophyra were closely correlated with port throughput. The change in Bacillariophyta was the most notable feature, followed by Haptophyta, Pyrrophyta, Ochrophyta, and Ciliophora.

## 3. Discussion

### 3.1. Increasing Dominance of HAB Species

New features of HABs in the Chinese coastal waters are that the area of the outbreak is enlarged, the duration is longer, the global expansion is obvious, and the disaster effect is aggravated [21]. The occurrence of HAB-related species in the Chinese coastal waters was caused frequently by dinoflagellates (Figure 4), which were harmful and last for a long time. Some dinoflagellates are able to survive fluctuations of environmental variables, exceeding the tolerance range for vegetative cells by producing resting cysts, and resume vegetative growth in favorable conditions [22]. Cysts formed and existed in the sea water or sediments when the HAB-related species subsided [23] and germinated under suitable environmental conditions. Furthermore, the causative species of HABs showed an increasing trend in China sea. More algal species may be found because of the level of development of biotechnology. Algae invasion caused by marine transport is also responsible for the increasing of algal species [24]. One of the important reasons for the frequent occurrence and geographical spread of HAB-related species in the world is that it spreads through ballast waters [25,26]. Changes in Ochrophyra were closely correlated with port throughput from 2002 to 2017, followed by Pyrrophyta (Figure 7). With the development of Chinese maritime transport industry, the throughput of Chinese coastal ports had increased year by year (Figure 6). The ballast water entering and leaving Chinese coastal waters reached billions of tons every year, which is the largest amount of ballast water in the world. The range of algae diffusion with ocean current is limited, but it spreads to the sea area tens of thousands of meters away through ballast water [26], and this may be the reason for the increase in harmful algal species. Most algae do not easily survive in ballast water, but cysts with strong survival ability can germinate rapidly under suitable environmental conditions after being discharged into other sea areas with ballast water [27]. For example, *Karenia mikimotoi* of Japanese origin was first discovered in Hong Kong in 1980, and *Heterosigma akashiwo* of Japanese origin was first discovered in Dalian Bay in 1995; now, they cause HABs all year round [28]. Therefore, the outbreak of alien algae caused by ballast water discharge and algae cysts in sea sediment should not be underestimated.

### 3.2. Relationship between River Pollutants Entering the Sea and HABs

Land-based pollutants, including industrial, domestic sewage, and agricultural non-point source pollution, increased every year and flowed into the ocean with the river and resulted in eutrophication of the sea area [29,30,31]. Compared with other sea areas, the eutrophication in the East China Sea was the most serious, which drove HABs in this area. *Noctiluca scintillans*, a causative species for HAB, mainly feed on phytoplankton organisms [32]. The high abundance of phytoplankton under eutrophic conditions led to a *Noctiluca scintillans* outbreak around the coast, especially in the Yangtze River Estuary [10]. Although the Yellow River contributes fewer pollutants to the Bohai Sea, the worst water exchange capacity in the Bohai Sea leads to severe pollution, supporting HABs in the area. Compared with the pollutants and water exchange capacity, the South China Sea is the opposite, but the small difference in sea surface temperature between different seasons supports the outbreak of HABs throughout the year. The pollutants entering the Yellow Sea are minimal, and active ocean currents in the area make HABs less severe. However, HAB-related species showed various response to nutrients. For example, diatoms always lead to the consumption of a large number of nutrients, especially silicate; and diatom bloom dissipates along with the depletion of silicate [33,34]. The toxic dinoflagellates *Prorocentrum donghainse* and *Karenia mikimotoi* are the main algae in the East China Sea in recent years and show different characteristic concentrations under different N/P ratios [35,36]. Additionally, some algae could form cysts to survive excessive oligotrophic condition, and their abundance could increase rapidly once nutrients nourished, leading to HAB breakout [37,38]. Therefore, the eutrophication of sea area is likely to be the important reasons for the outbreak of HAB-related species in China.

### 3.3. Potential Impacts of Hydrology and Meteorology on HABs

**Figure 8:**
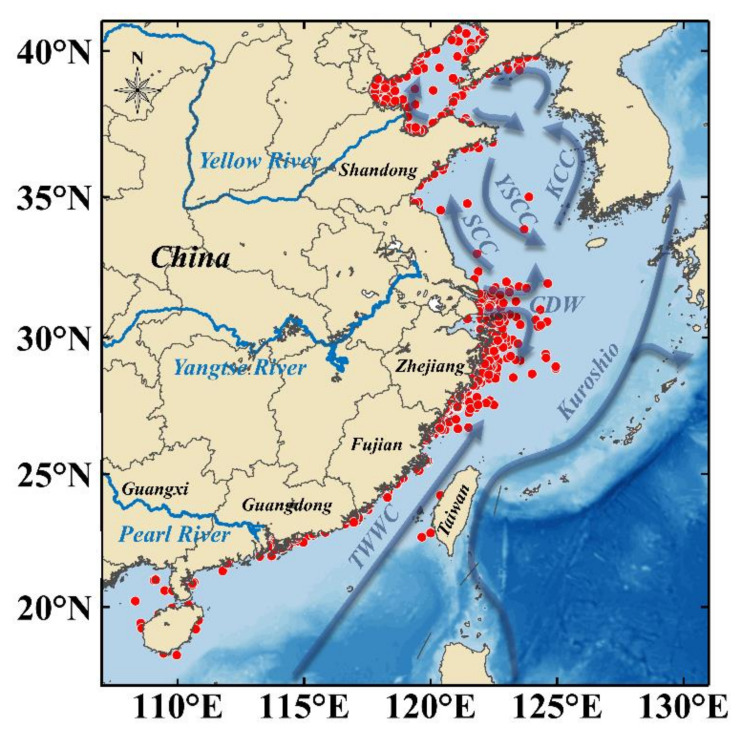
Main ocean currents and HABs locations (the blue arrow indicates the Taiwan Warm Current (TWWC), Changjiang Diluted Water (CDW), Yellow Sea Coastal current (YSCC), Subei Coastal Current (SCC), and Korea Coastal Current (KCC); red spots represent the location of HABs).

In addition to marine pollution or other human activities, meteorological and hydrological factors are also important [39]. The eutrophication of the East China Sea and the Yellow Sea is the highest in autumn, and the eutrophication area of the Bohai Sea is less in spring and summer [11], but HAB-related species occur in late spring and early summer, gradually decreasing in autumn. Sea surface temperature and sea surface salinity are closely related to HAB-related species and mainly drive Bacillariophyta, Haptophyte, and Ciliophora. 25 °C is suitable for the growth of almost Chinese algae [39]. The annual temperature of the South China Sea is between 25–28 °C, resulting in no obvious seasonal characteristic of HABs in the South China Sea. With the rapid deterioration of global climate, meteorological disasters are increasing, such as abnormal El Niños and typhoons. Previous studies reported that the variations in surface temperature caused by El Niños increased the probability of HAB-related species [12,40]. During the passage of a typhoon, the sea surface temperature drops by 0.2–4 °C [41], and salinity increases or decreases by 10 PSU [42], making the algae bloom. At the same time, the strong current caused by the typhoon makes the algal cysts in the sediments rise or migrate and then begin to germinate under appropriate environmental conditions; this may be why Bacillariophyta and Pyrrophyta are closely related to wind. There are many ocean currents in China, such as the Taiwan warm current, Yellow Sea current, and Kuroshio (Figure 8). Although the ocean current accelerates the speed of seawater purification, it also affects the heat transfer and exchange between surface and bottom layers, causing a maximum increase in sea temperature of 2.43° [43], which is a particularly contributing factor to the outbreak of HABs in nutrient-rich estuaries [17]. The upwelling compensation current brings the nutrients and algae in the deep ocean to surface, resulting in HABs. The most serious area for blooming of algae is located in the Yangtze River Estuary and adjacent waters in China [44]. The expansion direction and time area of nutrient-rich Changjiang diluted water varies from season to season, which extends northeast in summer and downward in autumn and winter [45]. Meanwhile, the confluence of the Taiwan warm current and the Changjiang diluted water creates suitable conditions for the growth of algae in certain places and seasons [46], leading to HABs [35,47]. The Yellow River Estuary of the Bohai Sea and the Pearl River Estuary of the South China Sea, similar to the hydrological conditions of the Yangtze River Estuary, are also the main sites of HABs [5,9,10,17]. Therefore, in addition to the pollution caused by pollutants discharged from rivers, attention should also be paid to the impacts of meteorological and hydrological conditions on algae.

### 3.4. Response and Prospect of HABs

Although the prevention and treatments of HABs started lately in China, the situation has been reduced in recent years, especially in the area of algal bloom (Figure 2). It reflects that the prevention and treatment of HABs have made great progress, such as physical catching [21] and adding modified clay [48,49]. At the same time, China has formulated various systems, laws, and regulations to prevent and control algal blooms. For example, the Red Tide Contingency Plan has further improved China’s HABs monitoring, early warning, and disaster investigation. Regulations on the Prevention of Damage to the Marine Environment by Land-based Pollutants from damaging the marine environment establishes the specific system for the implementation of environmental management [50], which effectively blocks the entry of land-based pollutants into the sea. Regulations on the Prevention of Marine Pollution by Ships regulates the discharge of pollutants used by ships, which is conducive to preventing HABs caused by foreign algae in ship ballast water [51]. There is no doubt that these measures and policies are effective in reducing HABs in China in recent years, but the discharge forms of industrial wastewater, domestic sewage, and aquaculture wastewater are still severe. In the future, China should continue to strengthen the control of land-based pollutants entering the sea, aquaculture pollution management, and the management of ship ballast water discharge.

## 4. Conclusions

Data on HABs from 1990 to 2017 in the Chinese coastal waters were collected and analyzed. Frequency and areas of HABs have slightly reduced after 2008, but they expanded over large coastal areas with high frequency and an increased diversity of HAB species. The frequency and affected area of HABs exhibited an obvious time- and space-dependent feature. The number of algal species increased since 2000; *Prorocentrum donghaiense*, *Noctiluca scientillans*, *Karenia mikimotoi*, and *Skeletonema costatum* were the most dominant algal species. Due to the control of land-based pollutants discharged into the sea, aquaculture wastewater, and ship ballast water, HABs showed a small decrease in recent years. HABs mainly occur in late spring and early summer in China. The sea surface temperature results from the currents may provide favorable conditions for the algae. A large number of dinoflagellate cysts exist in the sediment and are lifted by the upwelling, resulting in the occurrence of HABs. Algae may be transported by ballast water discharge, which may increase the algal species in China. Although HABs in China has been alleviated, it is still necessary to continue to strengthen the monitoring and prevention and enhance the control of the discharge of land-based pollutants into the sea, domestic sewage, aquaculture wastewater, and ship ballast water in the future.

## 5. Materials and Methods

### 5.1. Study Area

China is located in the east of the Asian continent, facing the Pacific Ocean. Bohai Sea (37° N~41° N, 117.5° E~121° E), Yellow Sea (35.5° N~36.75° N, 120° E~124° E), East China Sea (23° N~33.16° N, 117.16° E~131° E), and South China Sea (4° N~21° N, 105° E~118° E) are adjacent to the Chinese mainland and interconnected into one another (Figure 1a). The four seas cross temperate zone, subtropical zone, and tropical zone. Suspended solids and nutrients in Chinese coastal waters are significantly related by rivers, such as the Yellow River, the Yangtze River, and the Pearl River, which are rich in fishery resources [9,10,13,14,15].

### 5.2. Data Sources and Method

In this study, data on HABs and environmental variables were collected and compiled in the Chinese coastal waters (Table 1). The data were based on regularly issued reports from the State Oceanic Administration of China, including the annual Marine Environment Quality Bulletin [52] and China Marine Disaster Bulletin [53]. In addition, based on the existing data, some historical data were sourced from papers that provided information of the frequency and area of HABs in each sea water and related specie of HABs in the Chinese coastal waters [10,19]. The frequency, area, and related species of HABs were extracted digitally from the pictures or charts in the literature. The location and time of HABs were provided by the National Marine Environmental Monitoring Center. The data can be accessed in Supplementary Materials. Because the State Oceanic Administration of China began publishing HAB-related species in 2000, the analysis time was from 2000 to 2017. However, the loss of some historical data does not affect the trend of HABs in a certain area [54]. Besides, port throughput data come from the Ministry of Transport, PRC (https://www.mot.gov.cn/tongjishuju/gangkouhuowulvkettl/index_10.html (accessed on 21 September 2021)). The pollutant emissions data (chemical oxygen demand (COD), nutrients, and petroleum) come from the Marine Environment Quality Bulletin, and the data of wind speed, rainfall, sea surface temperature, and salinity come from the NCEP (National Centers for Environmental Prediction) data published by Asia-pacific data-research center (http://apdrc.soest.hawaii.edu/las/v6/dataset?catitem=16712 (accessed on 21 September 2021)).

### 5.3. Statistical Analysis

ArcGIS (version ArcGIS 10.2, Esri, RedLands, CA, USA, 2013) and Origin (version Origin2018, OriginLab, Northampton, MA, USA, 2018) were used to visualize data. Linear and non-linear regression models were used to fit the trend of environmental variables, including the frequency and area of HABs and the cargo throughput of Chinese ports. Matlab (version Matlab 2014b, MathWorks, Natick, MA, USA, 2014) was used for regression models. Principal component analysis of algal species and environmental factors was performed using Origin. Under the premise of eigenvalue >1, two principal components were extracted, which were used to investigate the drivers of HABs in the coastal China sea. In China, the criterion for judging HABs mainly depends on the abundance of the algal species. When the abundance (cell/dm^3^) of algal species was greater than 10^7^ (length of algae less than 10 μm), 10^6^ (length of algae between 10 μm and 29 μm), 3 × 10^5^ (length of algae between 30 μm and 99 μm), 10^5^ (length of algae between 100 μm and 299 μm), and 10^4^ (length of algae between 300 μm and 1000 μm), we judged it as HABs [9].

## Figures and Tables

**Table 1:** Sources of HABs and environmental variables.

Name	Period of Analysis	Source
Area Area (each sea water)	1990–2017 1995–2017	China Marine Disaster Bulletin Literatures [10,19]
Frequency Frequency (each sea water)	1990–2017 1995–2017	China Marine Disaster Bulletin Literatures [10,19]
Rainfall	2002–2017	National Centers for Environmental Prediction
Wind speed	2002–2017	National Centers for Environmental Prediction
Dominant species	2000–2017	China Marine Disaster Bulletin
Port throughput	1998–2017	Ministry of Transport, PRC
Pollutant emissions	2002–2017	Marine Environment Quality Bulletin
Sea surface salinity	2002–2017	National Centers for Environmental Prediction
Sea surface temperature	2002–2017	National Centers for Environmental Prediction

## Data Availability

The data presented in this study are available in Supplementary Materials.

## Supplementary Materials

The following supporting information can be downloaded at: https://www.mdpi.com/article/10.3390/toxins14030160/s1, Table S1: Recorded information on the frequency and scale of HABs in China coastal waters; Table S2: Recorded information on main causative groups of HABs in China coastal waters; Table S3: Recorded information on the total number and total area of HABs in the four areas of China.
