# Supplementary material for: Characteristics of Harmful Algal Species in the Coastal Waters of China from 1990 to 2017
Source: Toxins (Basel). 2022 Feb 23;14(3):160. doi: 10.3390/toxins14030160 (PMC8951513; doi:10.3390/toxins14030160)
Supplement: Supplementary file 1 [file toxins-14-00160-s001.zip › toxins-1486787-supplementary.pdf]

Supplementary Materials: Characteristics of harmful algal blooms in the coastal waters of China from 1990 to 2017

Wanli Hou, Xi Chen, Menglin Ba, Jianghua Yu, Tiantian Chen, Yihui Zhu, Jie Bai

Table S1. Recorded information on the frequency and scale of HABs in China coastal waters.

Start year	Total number	Total area (km^2)	Source
1990	34	16147	China Marine Disaster Bulletin
1991	38	511	China Marine Disaster Bulletin
1992	50	1596	China Marine Disaster Bulletin
1993	19	999	China Marine Disaster Bulletin
1994	12	69	China Marine Disaster Bulletin
1995	16	1416	China Marine Disaster Bulletin
1996	12	4371	China Marine Disaster Bulletin
1997	8	64	China Marine Disaster Bulletin
1998	22	7263	China Marine Disaster Bulletin
1999	16	9365	China Marine Disaster Bulletin
2000	28	10650	China Marine Disaster Bulletin
2001	77	15000	China Marine Disaster Bulletin
2002	79	10000	China Marine Disaster Bulletin
2003	119	14550	China Marine Disaster Bulletin
2004	96	26630	China Marine Disaster Bulletin
2005	82	27070	China Marine Disaster Bulletin
2006	93	14970	China Marine Disaster Bulletin
2007	82	11610	China Marine Disaster Bulletin
2008	68	13738	China Marine Disaster Bulletin
2009	68	14102	China Marine Disaster Bulletin
2010	69	10892	China Marine Disaster Bulletin
2011	55	6076	China Marine Disaster Bulletin

2012	73	7971	China Marine Disaster Bulletin
2013	46	4070	China Marine Disaster Bulletin
2014	56	7290	China Marine Disaster Bulletin
2015	35	2809	China Marine Disaster Bulletin
2016	68	7484	China Marine Disaster Bulletin
2017	68	3679	China Marine Disaster Bulletin

Table S2. Recorded information on main causative groups of HABs in China coastal waters.

Start year	Distribution area	Longitude (°E)	Latitude (°N)	Major causative species	Source
1980	South China Sea	114.2003	22.2866	-	NMEMC
1980.5.17	South China Sea	110.4297	21.1200	*Leptocylindrus danicus*	NMEMC
1980.9	South China Sea	114.3667	22.4500	*Noctiluca scintillans*	NMEMC
1981.3.01	South China Sea	113.9397	22.4845	*Gymnodinium aeruginosum*	NMEMC
1981.6.16	East China Sea	122.3018	29.0701	*Noctiluca scintillans*	NMEMC
1981.8	Yellow Sea	121.6800	39.0000	*Dactyliosolen fragilissimus*	NMEMC
1981.8.15	East China Sea	122.2133	28.6433	*Noctiluca scintillans*	NMEMC
1981.9.28	East China Sea	120.2741	26.9400	*Noctiluca scintillans*	NMEMC
1982.11.01	South China Sea	113.5239	22.2186	*Gymnodinium aeruginosum*	NMEMC
1982.3	South China Sea	113.5239	22.2186	*Gymnodinium aeruginosum*	NMEMC
1982.6.16	East China Sea	120.8463	27.9751	-	NMEMC
1982.8.20	East China Sea	122.3564	30.0429	*Noctiluca scintillans*	NMEMC
1982.8.24	East China Sea	122.6186	31.0129	*Noctiluca scintillans*	NMEMC
1982.8-9	East China Sea	122.4517	31.0403	*Noctiluca scintillans*	NMEMC
1983	South China Sea	114.1925	22.2928	-	NMEMC
1983.3	East China Sea	121.6500	29.1400	*Gymnodinium aeruginosum*	NMEMC
1983.4	South China Sea	114.6386	22.5583	*Rhizosolenia fragilissima*	NMEMC
1984	South China Sea	114.2056	22.2784	-	NMEMC
1984.10.2	East China Sea	120.1946	26.8972	*Noctiluca scintillans*	NMEMC
1984.4	South China Sea	114.3686	22.3076	*Rhizosolenia fragilissima*	NMEMC
1984.6.9	Yellow Sea	122.9887	39.6545	-	NMEMC
1984.7.16	South China Sea	113.7809	22.0410	*Trichodesmium contortum*	NMEMC
1984.7.27	South China Sea	109.4926	20.6274	*Trichodesmium contortum*	NMEMC
1984.7.8	Yellow Sea	121.6840	38.9661	*Scrippsiella trochoidea*	NMEMC
1985.5.9-19	South China Sea	113.9238	22.4697	*Pseudo-nitzschia pungens*	NMEMC

1985.6.3	South China Sea	109.1273	21.0603	*Trichodesmium contortum*	NMEMC
1985.7	South China Sea	113.7809	22.0410	*Pseudo-nitzschia pungens*	NMEMC
1985.7.12	Yellow Sea	121.6594	38.9917	*Heterosigma akashiwo*	NMEMC
1985.8.28	Yellow Sea	121.6512	38.9981	*Skeletonema costatum*	NMEMC
1986	South China Sea	114.1835	22.3011	-	NMEMC
1986.1	East China Sea	120.0000	22.7900	*Thalassiosira rotula*	NMEMC
1986.11.2	East China Sea	117.5522	23.8751	*Gymnodinium aeruginosum*	NMEMC
1986.2	South China Sea	113.9418	22.4845	*Noctiluca scintillans*	NMEMC
1986.4	South China Sea	113.9238	22.4550	*Gymnodinium aeruginosum*	NMEMC
1986.4.23	Yellow Sea	121.5855	38.9468	*Thalassiosira nordenskioeldii*	NMEMC
1986.5.17	East China Sea	118.0600	24.4400	*Leptocylindrus danicus*	NMEMC
1986.5.24	East China Sea	122.4120	29.1957	*Noctiluca scintillans*	NMEMC
1986.6.1	East China Sea	122.3802	31.2864	*Noctiluca scintillans*	NMEMC
1986.6.18	Yellow Sea	121.6512	38.9661	*Skeletonema costatum*	NMEMC
1986.6.20	East China Sea	118.0645	24.4590	*Cochlodinium polykrikoides*	NMEMC
1986.6.7-8	East China Sea	122.3802	30.9856	*Noctiluca scintillans*	NMEMC
1986.7.22	Yellow Sea	121.6840	38.9853	*Skeletonema costatum*	NMEMC
1986.7.8-13	Yellow Sea	121.7168	38.9533	*Heterosigma akashiwo*	NMEMC
1987.10	South China Sea	114.3844	22.4845	*Noctiluca scintillans*	NMEMC
1987.10	South China Sea	114.6800	22.6600	*Trichodesmium contortum*	NMEMC
1987.10	East China Sea	122.0100	29.2300	*Noctiluca scintillans*	NMEMC
1987.2-5	South China Sea	114.3500	22.4500	*Gonyaulax polygramma*	NMEMC
1987.3.17	East China Sea	118.0600	24.4400	*Chaetoceros socialis*	NMEMC
1987.4	South China Sea	113.8205	22.4550	*Skeletonema costatum*	NMEMC
1987.4	South China Sea	113.8217	22.4550	*Gymnodinium aeruginosum*	NMEMC
1987.4.17	East China Sea	118.0600	24.4400	*Nitzschia delicatissima*	NMEMC
1987.5.1	East China Sea	118.1110	24.4164	*Skeletonema costatum*	NMEMC
1987.5.11	East China Sea	118.0600	24.4400	*Leptocylindrus danicus*	NMEMC
1987.6.11	East China Sea	122.4363	31.1488	*Noctiluca scintillans*	NMEMC

1987.6.18	Yellow Sea	121.6594	38.9725	*Heterosigma akashiwo*	NMEMC
1987.6.30	East China Sea	122.4363	31.1488	*Skeletonema costatum*	NMEMC
1987.7.1	South China Sea	114.1969	22.3011	-	NMEMC
1987.7.1	East China Sea	122.8085	30.9084	*Skeletonema costatum*	NMEMC
1987.7.20	Yellow Sea	121.6594	38.9917	*Skeletonema costatum*	NMEMC
1987.7.8	East China Sea	122.0000	29.0400	*Skeletonema costatum*	NMEMC
1987.8.1	East China Sea	122.3742	30.7745	-	NMEMC
1987.8.1	East China Sea	122.6224	31.4418	*Noctiluca scintillans*	NMEMC
1987.8.14	South China Sea	114.6544	22.4697	*Trichodesmium contortum*	NMEMC
1987.8.14	East China Sea	122.0900	31.0800	*Noctiluca scintillans*	NMEMC
1988.12.1	South China Sea	114.2733	22.5435	*Noctiluca scintillans*	NMEMC
1988.12.2	South China Sea	114.3844	22.4845	*Noctiluca scintillans*	NMEMC
1988.2.15	South China Sea	114.3844	22.4845	*Noctiluca scintillans*	NMEMC
1988.3.14	South China Sea	114.6800	22.6600	*Noctiluca scintillans*	NMEMC
1988.4.1	South China Sea	113.9397	22.5140	*Noctiluca scintillans*	NMEMC
1988.6.1	East China Sea	122.4053	31.1221	*Noctiluca scintillans*	NMEMC
1988.6.13	East China Sea	122.4363	31.2821	*Noctiluca scintillans*	NMEMC
1988.7.17	East China Sea	122.4363	31.2821	*Noctiluca scintillans*	NMEMC
1988.7-8	East China Sea	122.0900	31.0800	*Noctiluca scintillans*	NMEMC
1988.8.1	East China Sea	121.8810	32.3717	-	NMEMC
1988.8.22	East China Sea	122.0900	31.0800	*Noctiluca scintillans*	NMEMC
1989.10.1	East China Sea	117.2274	23.6162	*Scrippsiella trochoidea*	NMEMC
1989.11.2	East China Sea	122.1900	30.9900	-	NMEMC
1989.2	South China Sea	114.4003	22.5435	*Noctiluca scintillans*	NMEMC
1989.3.1	South China Sea	114.4003	22.5435	*Noctiluca scintillans*	NMEMC
1989.4.1	South China Sea	114.3844	22.4845	*Noctiluca scintillans*	NMEMC
1989.4.1	Yellow Sea	121.6184	38.9533	*Thalassiosira nordenskioeldii*	NMEMC
1989.4.17	South China Sea	114.4003	22.5435	*Noctiluca scintillans*	NMEMC
1989.4.20	East China Sea	120.8804	27.9572	*Noctiluca scintillans*	NMEMC

1989.4.22	East China Sea	119.5173	25.6341	*Noctiluca scintillans*	NMEMC
1989.4.2-4.4	South China Sea	113.9238	22.4697	*Skeletonema costatum*	NMEMC
1989.4.29	East China Sea	117.3100	23.6600	*Skeletonema costatum*	NMEMC
1989.4.30	South China Sea	108.6300	19.3200	*Pseudo-nitzschia pungens*	NMEMC
1989.4.7-4.10	South China Sea	113.8600	22.4400	*Noctiluca scintillans*	NMEMC
1989.5.1	East China Sea	118.0533	24.4477	*Leptocylindrus danicus*	NMEMC
1989.5.6	East China Sea	120.1000	26.8500	*Gymnodinium aeruginosum*	NMEMC
1989.6.1	East China Sea	118.0533	24.4913	*Gymnodinium aeruginosum*	NMEMC
1989.7.1	Yellow Sea	121.5938	38.9468	*Heterosigma akashiwo*	NMEMC
1989.7.1	East China Sea	122.4210	30.9179	*Skeletonema costatum*	NMEMC
1989.7.13	East China Sea	122.0081	30.2308	*Skeletonema costatum*	NMEMC
1989.7.5-10	East China Sea	117.4974	23.6894	*Cochlodinium polykrikoides*	NMEMC
1989.7-11	East China Sea	122.0875	31.3142	-	NMEMC
1989.8.28	Yellow Sea	121.7004	38.9853	*Skeletonema costatum*	NMEMC
1989.8.4-10.15	Bohai Sea	117.6878	38.5076	*Pseudo-nitzschia pungens*	NMEMC
1989.8-9	Bohai Sea	119.2578	37.3398	*Noctiluca scintillans*	NMEMC
1989.9.22	East China Sea	121.3611	28.0981	-	NMEMC
1990.1.26	South China Sea	114.2097	22.4550	-	NMEMC
1990.10.1	East China Sea	122.5322	30.8083	-	NMEMC
1990.10.4	East China Sea	122.5640	31.4368	-	NMEMC
1990.11.2	South China Sea	114.3844	22.4845	*Dactyliosolen fragilissimus*	NMEMC
1990.11.6	South China Sea	114.3844	22.4845	*Dactyliosolen fragilissimus*	NMEMC
1990.12.2	South China Sea	114.3844	22.4845	*Pseudo-nitzschia pungens*	NMEMC
1990.12.5	South China Sea	114.3844	22.4845	*Pseudo-nitzschia pungens*	NMEMC
1990.2.13	South China Sea	114.3844	22.4845	*Noctiluca scintillans*	NMEMC
1990.3.10	South China Sea	114.3844	22.4845	*Noctiluca scintillans*	NMEMC
1990.3.18	South China Sea	114.3686	22.3076	*Noctiluca scintillans*	NMEMC
1990.3.20	South China Sea	114.3844	22.4845	*Noctiluca scintillans*	NMEMC
1990.3.30	South China Sea	114.3844	22.4845	*Noctiluca scintillans*	NMEMC

1990.4.1-2	South China Sea	114.2891	22.4845	-	NMEMC
1990.4.15-17	South China Sea	113.7333	22.4107	-	NMEMC
1990.4.18	South China Sea	114.3209	22.5583	*Noctiluca scintillans*	NMEMC
1990.4.20	South China Sea	114.2891	22.5435	*Noctiluca scintillans*	NMEMC
1990.4.20	East China Sea	120.9000	27.9300	*Noctiluca scintillans*	NMEMC
1990.4.29	East China Sea	117.3227	23.6601	*Skeletonema costatum*	NMEMC
1990.4.30	South China Sea	108.5397	19.3813	*Chaetoceros compressus*	NMEMC
1990.4.7-10	South China Sea	113.7333	22.4107	*Noctiluca scintillans*	NMEMC
1990.5.1	East China Sea	121.0075	27.4766	-	NMEMC
1990.5.10	South China Sea	114.3844	22.4845	*Noctiluca scintillans*	NMEMC
1990.5.1-9	East China Sea	120.1816	26.8943	-	NMEMC
1990.5.20-27	Bohai Sea	117.6000	38.6167	*Pseudo-nitzschia pungens*	NMEMC
1990.5.21	East China Sea	121.8969	31.2869	-	NMEMC
1990.5.6	East China Sea	120.0617	26.8495	*Gymnodinium aeruginosum*	NMEMC
1990.5.9-11	East China Sea	121.4522	28.0277	-	NMEMC
1990.6	South China Sea	114.3844	22.4845	*Noctiluca scintillans*	NMEMC
1990.6	Bohai Sea	119.2004	39.3303	-	NMEMC
1990.6.10	South China Sea	114.3844	22.4845	*Pseudo-nitzschia pungens*	NMEMC
1990.6.12	Yellow Sea	120.9865	38.4453	*Noctiluca scintillans*	NMEMC
1990.6.12	Yellow Sea	121.3700	38.7900	*Noctiluca scintillans*	NMEMC
1990.6.14	East China Sea	121.9287	31.0684	-	NMEMC
1990.6.18	Bohai Sea	119.4493	37.3245	*Noctiluca scintillans*	NMEMC
1990.6.21-22	Bohai Sea	117.6878	38.5678	*Noctiluca scintillans*	NMEMC
1990.6.24	Yellow Sea	120.2726	36.1378	*Mesodiniu rubrum*	NMEMC
1990.6.30	East China Sea	121.9287	31.0684	-	NMEMC
1990.6.3-7	East China Sea	121.8403	31.2050	-	NMEMC
1990.6.9-14	East China Sea	122.5004	30.9590	*Skeletonema costatum*	NMEMC
1990.7	Bohai Sea	118.5676	38.1859	-	NMEMC
1990.7	Bohai Sea	119.0089	37.4009	-	NMEMC

1990.7	Bohai Sea	119.2195	39.3303	*Noctiluca scintillans*	NMEMC
1990.7.1	Yellow Sea	121.5773	38.8507	*Noctiluca scintillans*	NMEMC
1990.7.10	East China Sea	121.6428	29.0654	-	NMEMC
1990.7.13	East China Sea	121.9763	29.0654	-	NMEMC
1990.7.26	East China Sea	122.2781	30.1618	-	NMEMC
1990.7.26	East China Sea	122.3257	31.4232	-	NMEMC
1990.7.26	East China Sea	122.3734	31.4777	-	NMEMC
1990.7.3-5	East China Sea	122.3416	30.7671	-	NMEMC
1990.7.4	East China Sea	122.2622	31.1777	-	NMEMC
1990.7.5-9	Yellow Sea	121.6430	38.9661	*Heterosigma akashiwo*	NMEMC
1990.7.8-10	Yellow Sea	121.7250	39.0173	*Heterosigma akashiwo*	NMEMC
1990.8.1	Yellow Sea	122.4143	39.3175	-	NMEMC
1990.8.13-28	Bohai Sea	117.7000	38.3000	*Chaetoceros compressus*	NMEMC
1990.8.19	Bohai Sea	119.0472	37.3704	-	NMEMC
1990.8.2	East China Sea	122.5163	30.7946	-	NMEMC
1990.8.26	Bohai Sea	119.7174	37.3398	-	NMEMC
1990.8.30	Bohai Sea	119.5642	37.2939	-	NMEMC
1990.8.31	Yellow Sea	121.6676	38.9917	*Pseudo-nitzschia pungens*	NMEMC
1990.8.6-7	East China Sea	122.1669	30.9727	-	NMEMC
1990.8.6-7	East China Sea	122.3098	30.6848	-	NMEMC
1990.9.10-20	Bohai Sea	117.6833	38.4833	*Noctiluca scintillans*	NMEMC
1990.9.20	East China Sea	122.4369	31.4232	-	NMEMC
1990.9.27	East China Sea	122.7017	30.9162	-	NMEMC
1991.1.28-2.3	South China Sea	109.7785	19.9680	*Noctiluca scintillans*	NMEMC
1991.1.5	South China Sea	114.3844	22.4845	*Pseudo-nitzschia pungens*	NMEMC
1991.10	Bohai Sea	117.7644	38.8977	*Nitzschia closterium*	NMEMC
1991.12.2	South China Sea	114.2256	22.4402	*Noctiluca scintillans*	NMEMC
1991.2	South China Sea	114.3844	22.4845	*Noctiluca scintillans*	NMEMC
1991.2.1	South China Sea	120.4039	24.2003	-	NMEMC

1991.2.24	South China Sea	114.3844	22.4845	*Gymnodinium aeruginosum*	NMEMC
1991.2.4	South China Sea	108.5873	19.1852	*Noctiluca scintillans*	NMEMC
1991.3.1	South China Sea	113.8762	22.4550	*Noctiluca scintillans*	NMEMC
1991.3.10	South China Sea	114.3844	22.4845	*Pseudo-nitzschia pungens*	NMEMC
1991.3.11	South China Sea	109.2544	19.7276	*Prorocentrum donghaiense*	NMEMC
1991.3.19	South China Sea	114.2574	22.5435	*Protoperidinium bipes*	NMEMC
1991.3.25	South China Sea	114.3050	22.5583	*Noctiluca scintillans*	NMEMC
1991.3.7	South China Sea	114.2733	22.5140	*Noctiluca scintillans*	NMEMC
1991.4.17	South China Sea	114.3686	22.5140	*Noctiluca scintillans*	NMEMC
1991.4.9	South China Sea	114.3844	22.4845	*Noctiluca scintillans*	NMEMC
1991.5.10	East China Sea	120.4600	27.1500	*Prorocentrum donghaiense*	NMEMC
1991.5.12	East China Sea	120.4100	26.6700	-	NMEMC
1991.5.12	East China Sea	120.4100	26.6700	-	NMEMC
1991.5.19	South China Sea	120.4028	27.0315	*Prorocentrum donghaiense*	NMEMC
1991.5.5-15	South China Sea	110.7156	19.1551	*Gonyaulax polygramma*	NMEMC
1991.5.5-6	South China Sea	114.2891	22.5140	*Gonyaulax polygramma*	NMEMC
1991.5-7	Bohai Sea	121.1725	38.9725	-	NMEMC
1991.5-7	Bohai Sea	121.1917	38.9725	-	NMEMC
1991.5-7	Bohai Sea	121.7852	40.8885	-	NMEMC
1991.6.2	South China Sea	114.3844	22.4845	*Gonyaulax polygramma*	NMEMC
1991.6.8-16	East China Sea	121.3100	28.1400	*Noctiluca scintillans*	NMEMC
1991.7.1	Yellow Sea	121.5938	38.8571	*Skeletonema costatum*	NMEMC
1991.7.10-16	Bohai Sea	117.7250	38.4083	*Chaetoceros compressus*	NMEMC
1991.7.17-24	Bohai Sea	117.6833	38.4833	*Noctiluca scintillans*	NMEMC
1991.7.4-12	Bohai Sea	121.9575	40.2461	*Noctiluca scintillans*	NMEMC
1991.7.6	South China Sea	114.3844	22.4845	*Rhizosolenia fragilissima*	NMEMC
1991.8	Bohai Sea	122.0150	40.2461	*Noctiluca scintillans*	NMEMC
1991.8.1	Yellow Sea	121.5527	38.8507	*Skeletonema costatum*	NMEMC
1991.8.17	Bohai Sea	117.6500	38.5000	*Cochlodinium polykrikoides*	NMEMC

1991.8.17	Bohai Sea	117.6500	38.5000	*Cochlodinium polykrikoides*	NMEMC
1991.8.17-19	Bohai Sea	117.6667	38.4917	*Protoperidinium bipes*	NMEMC
1991.8.2	East China Sea	122.0700	30.5200	-	NMEMC
1991.8.20-22	Bohai Sea	117.6667	38.4917	*gymnodinium-mikimotoi*	NMEMC
1991.8.20-9.6	Bohai Sea	117.7333	38.5500	*Ceratium trichoceros*	NMEMC
1991.8.30-9.2	Bohai Sea	117.6500	38.5000	*Protoperidinium bipes*	NMEMC
1991.8.30-9.2	Bohai Sea	117.6667	38.4917	*Protoperidinium bipes*	NMEMC
1991.9	Bohai Sea	122.0724	40.4365	-	NMEMC
1991.9.14	Bohai Sea	117.6250	38.5583	*Mesodiniu rubrum*	NMEMC
1991.9.17	South China Sea	114.3686	22.5583	*Noctiluca scintillans*	NMEMC
1991.9.20-27	Bohai Sea	117.5917	38.5917	*Nitzschia closterium*	NMEMC
1991.9.20-27	Bohai Sea	117.7250	38.4083	*gymnodinium-mikimotoi*	NMEMC
1991.9.4	South China Sea	114.3844	22.4845	*Rhizosolenia fragilissima*	NMEMC
1991.9.4	Bohai Sea	117.6250	38.5583	*Thalassiosira rotula*	NMEMC
1992.10.2	South China Sea	114.3844	22.4845	*Alexandrium tamarense*	NMEMC
1992.11.3	South China Sea	114.3844	22.4845	*Rhizosolenia fragilissima*	NMEMC
1992.4.1	Yellow Sea	119.6982	35.6035	*Noctiluca scintillans*	NMEMC
1992.4.1	Yellow Sea	119.9362	35.6839	-	NMEMC
1992.4.21	South China Sea	114.3844	22.4845	*Noctiluca scintillans*	NMEMC
1992.4.24	South China Sea	114.3844	22.4845	*Noctiluca scintillans*	NMEMC
1992.4.27	South China Sea	114.7900	22.4900	*Noctiluca scintillans*	NMEMC
1992.4.29	South China Sea	114.3844	22.4845	*Noctiluca scintillans*	NMEMC
1992.4.6	South China Sea	109.8000	20.6167	*Chaetoceros socialis*	NMEMC
1992.5.1	Yellow Sea	123.8833	35.0000	*Noctiluca scintillans*	NMEMC
1992.5.5	South China Sea	114.3844	22.4845	*Noctiluca scintillans*	NMEMC
1992.5.8-23	East China Sea	122.1900	30.7800	*Noctiluca scintillans*	NMEMC
1992.6.5	East China Sea	121.0800	27.6400	*Noctiluca scintillans*	NMEMC
1992.7.1	Yellow Sea	121.5609	38.8507	*Skeletonema costatum*	NMEMC
1992.7.1	Yellow Sea	121.5773	38.8507	*Skeletonema costatum*	NMEMC

1992.7.16	Bohai Sea	117.5833	38.5667	*Gymnodinium aeruginosum*	NMEMC
1992.7.18-26	Bohai Sea	117.6250	38.5583	*Leptocylindrus danicus*	NMEMC
1992.7.21	Bohai Sea	117.6500	38.5000	*Skeletonema costatum*	NMEMC
1992.7.29	East China Sea	121.7200	29.1100	-	NMEMC
1992.8	South China Sea	114.4200	22.3600	-	NMEMC
1992.8.17-28	Bohai Sea	117.6167	38.5583	*gymnodinium-mikimotoi*	NMEMC
1992.8.20	East China Sea	122.6667	30.7167	-	NMEMC
1992.8.20	East China Sea	122.8200	30.7200	-	NMEMC
1992.8.22	East China Sea	122.4900	30.7500	-	NMEMC
1992.8.31-9.5	Bohai Sea	117.6167	38.5583	*Ceratium trichoceros*	NMEMC
1992.9.27	Bohai Sea	117.5833	38.5667	*gymnodinium-mikimotoi*	NMEMC
1992.9.5	Bohai Sea	117.6000	38.6167	*Ceratium trichoceros*	NMEMC
1993.4	South China Sea	114.3844	22.4845	*Noctiluca scintillans*	NMEMC
1993.6.17	East China Sea	122.4900	30.7500	-	NMEMC
1993.7.12	East China Sea	122.4900	30.7500	-	NMEMC
1993.8.15	Yellow Sea	122.2830	39.2282	*Chattonella marina*	NMEMC
1993.8.19	Yellow Sea	122.2830	39.2282	*Pseudo-nitzschia pungens*	NMEMC
1993.8.28	East China Sea	122.6700	30.8700	-	NMEMC
1994.5	South China Sea	113.8700	22.2700	*Noctiluca scintillans*	NMEMC
1994.5.1	Yellow Sea	122.5702	39.3175	*Noctiluca scintillans*	NMEMC
1994.6.2	East China Sea	122.4900	30.7500	-	NMEMC
1994.7.30	East China Sea	122.4900	30.7500	-	NMEMC
1994.8.1	Yellow Sea	121.4625	37.4773	*Chaetoceros siamense*	NMEMC
1995.1	Bohai Sea	120.1496	37.5883	*Ceratium trichoceros*	NMEMC
1995.2	Bohai Sea	120.8880	38.3678	*Noctiluca scintillans*	NMEMC
1995.3	South China Sea	114.3844	22.4845	*Noctiluca scintillans*	NMEMC
1995.5	Bohai Sea	120.0675	37.5622	*Noctiluca scintillans*	NMEMC
1995.5.17	East China Sea	121.6904	28.2670	-	NMEMC
1995.5.22	East China Sea	122.5640	31.8038	-	NMEMC

1995.5.27	East China Sea	122.5481	30.8220	-	NMEMC
1995.6.6	Bohai Sea	119.1700	37.4000	*Noctiluca scintillans*	NMEMC
1995.6.6	East China Sea	123.0722	29.7469	-	NMEMC
1995.8	Bohai Sea	119.9690	39.9836	*Noctiluca scintillans*	NMEMC
1995.8.15	Yellow Sea	122.3651	39.3876	*Chattonella marina*	NMEMC
1995.9.3	East China Sea	122.2600	31.0500	-	NMEMC
1996.4.26	South China Sea	113.9397	22.4697	-	NMEMC
1996.5	Yellow Sea	121.4700	37.6200	-	NMEMC
1996.5.22	East China Sea	123.2787	29.8715	-	NMEMC
1996.5.22	East China Sea	123.5487	29.1770	-	NMEMC
1996.5.8	Yellow Sea	121.3886	37.7123	-	NMEMC
1996.6.14	East China Sea	122.3892	31.6273	-	NMEMC
1996.6.26	Bohai Sea	117.7863	38.5806	-	NMEMC
1996.6.30	Bohai Sea	119.6244	39.8762	-	NMEMC
1996.9.18	Bohai Sea	117.9422	38.6322	-	NMEMC
1997.10.20	East China Sea	118.0600	24.4400	*Cochlodinium polykrikoides*	NMEMC
1997.10-12	South China Sea	116.9257	23.5284	*Phaeocystis scrobiculata*	NMEMC
1997.12	East China Sea	118.6900	24.8400	*Phaeocystis scrobiculata*	NMEMC
1997.12-1998.1	South China Sea	117.0527	23.6016	*Phaeocystis scrobiculata*	NMEMC
1997.4.13	Bohai Sea	120.7485	37.8621	-	NMEMC
1997.4.13	Yellow Sea	120.7600	37.8700	*Noctiluca scintillans*	NMEMC
1997.4.27	Bohai Sea	120.6665	37.9401	-	NMEMC
1997.5.18	East China Sea	123.5487	29.1770	-	NMEMC
1997.5.23	East China Sea	122.1200	31.5700	-	NMEMC
1997.5.31	East China Sea	122.1200	31.5700	-	NMEMC
1997.6.28	Bohai Sea	117.7534	38.3742	-	NMEMC
1997.7.28	East China Sea	122.6100	30.7500	*Noctiluca scintillans*	NMEMC
1998.10.1	Bohai Sea	119.4110	37.4053	-	NMEMC
1998.10.3	Bohai Sea	117.7534	38.9148	-	NMEMC

1998.10.9	Bohai Sea	118.3300	38.9600	-	NMEMC
1998.11.01	East China Sea	122.2700	31.2500	-	NMEMC
1998.11.1	South China Sea	114.6386	22.6025	*Mesodiniu rubrum*	NMEMC
1998.11.1	South China Sea	114.6800	22.6600	*Noctiluca scintillans*	NMEMC
1998.11.6	South China Sea	116.5921	23.1617	-	NMEMC
1998.11.7	South China Sea	115.1150	22.6762	-	NMEMC
1998.11.8	South China Sea	114.6800	22.6600	*Mesodiniu rubrum*	NMEMC
1998.1-2	South China Sea	115.3056	22.7499	*Phaeocystis scrobiculata*	NMEMC
1998.3.15	South China Sea	113.6856	22.2481	*Gymnodinium aeruginosum*	NMEMC
1998.3.15-4.15	South China Sea	113.6062	22.1594	*Karenia mikimotoi*	NMEMC
1998.4.20	South China Sea	112.0197	21.7294	*Karenia mikimotoi*	NMEMC
1998.4.20	South China Sea	113.8603	22.4402	*Pseudo-nitzschia pungens*	NMEMC
1998.4.23	South China Sea	113.8921	22.4550	*Pseudo-nitzschia pungens*	NMEMC
1998.5.18	South China Sea	114.5909	22.6762	*Skeletonema costatum*	NMEMC
1998.5.30	East China Sea	122.2700	31.2500	-	NMEMC
1998.6.3	East China Sea	122.2700	31.2500	-	NMEMC
1998.7.28	East China Sea	122.2700	31.2500	-	NMEMC
1998.7.3-8	Yellow Sea	120.2600	36.1500	*Skeletonema costatum*	NMEMC
1998.8.15	Bohai Sea	120.9119	40.4741	-	NMEMC
1998.8.15	Yellow Sea	121.4300	37.5700	*Gymnodinium aeruginosum*	NMEMC
1998.8.15	Bohai Sea	121.5000	37.5100	-	NMEMC
1998.8.24	Bohai Sea	120.7896	40.5681	-	NMEMC
1998.9.10	South China Sea	114.6544	22.7204	*Scrippsiella trochoidea*	NMEMC
1998.9.16	Bohai Sea	121.1000	40.3200	*Ceratium trichoceros*	NMEMC
1998.9.2	Bohai Sea	119.4192	37.3201	-	NMEMC
1998.9.22	Bohai Sea	120.4531	37.7905	-	NMEMC
1998.9.24	Bohai Sea	118.6889	39.0557	-	NMEMC
1998.9.26	Bohai Sea	119.4438	37.1495	*Ceratium trichoceros*	NMEMC
1998.9.29	Bohai Sea	121.0100	40.7000	*Ceratium trichoceros*	NMEMC

1999.3.14	South China Sea	114.3844	22.4845	*Gonyaulax polygramma*	NMEMC
1999.5.14	East China Sea	122.7228	30.5337	-	NMEMC
1999.5.15	East China Sea	123.1198	31.7495	-	NMEMC
1999.5.18	South China Sea	114.5542	22.7277	-	NMEMC
1999.5.20	South China Sea	114.3844	22.4845	*Cochlodinium polykrikoides*	NMEMC
1999.7.10	South China Sea	117.1333	23.6000	*Phaeocystis globosa*	NMEMC
1999.7.13	Bohai Sea	122.0122	40.3110	*Noctiluca scintillans*	NMEMC
1999.7.16	Bohai Sea	120.8716	38.3678	*Noctiluca scintillans*	NMEMC
1999.7.16	Bohai Sea	120.9701	39.0045	*Noctiluca scintillans*	NMEMC
1999.7.17	Bohai Sea	120.8833	39.0833	*Noctiluca scintillans*	NMEMC
1999.7.18	Bohai Sea	121.0029	38.4646	*Noctiluca scintillans*	NMEMC
1999.7.18	Yellow Sea	121.5199	38.8057	-	NMEMC
1999.7.18	Yellow Sea	121.6400	38.8500	*Noctiluca scintillans*	NMEMC
1999.7.2	Bohai Sea	118.0242	39.0173	-	NMEMC
1999.7.2-3	Bohai Sea	117.5647	38.6257	-	NMEMC
1999.7.24	Yellow Sea	120.2800	36.0800	-	NMEMC
1999.7.26	Yellow Sea	120.4300	36.0500	*Mesodiniu rubrum*	NMEMC
1999.7.26	Yellow Sea	120.4300	36.0500	*Mesodiniu rubrum*	NMEMC
1999.7.4	Bohai Sea	118.8100	38.1900	-	NMEMC
1999.8.6-7	Yellow Sea	122.4500	36.8900	-	NMEMC
1999.9	East China Sea	120.2026	26.8402	*Trichodesmium contortum*	NMEMC
1999.9.25	Yellow Sea	119.3777	38.7156	-	NMEMC
2000.05.03-04	East China Sea	123.3860	30.7500	-	NMEMC
2000.06.07-08	South China Sea	116.9500	23.4333	*Noctiluca scintillans*	NMEMC
2000.08.08-20	South China Sea	114.7333	22.6000	*Scrippsiella trochoidea*	NMEMC
2000.08.17	South China Sea	114.6083	22.6917	*Mesodiniu rubrum*	NMEMC
2000.09.3	South China Sea	114.7333	22.6000	*Scrippsiella trochoidea*	NMEMC
2000.1.12	South China Sea	113.7000	22.0167	*Mesodiniu rubrum*	NMEMC
2000.3.28	South China Sea	111.7833	21.3600	*Prorocentrum donghaiense*	NMEMC

2000.4.25	Bohai Sea	119.2400	39.3700	-	NMEMC
2000.5.12	East China Sea	121.8300	28.4700	-	NMEMC
2000.5.12	East China Sea	121.8300	28.4700	-	NMEMC
2000.5.18	East China Sea	122.5000	30.0150	*Prorocentrumdentatum*	NMEMC
2000.5.19	East China Sea	120.6100	27.1500	-	NMEMC
2000.5.20	East China Sea	122.6000	29.7333	-	NMEMC
2000.5.30	East China Sea	121.6500	29.0500	-	NMEMC
2000.5.30	East China Sea	121.6666	29.5833	-	NMEMC
2000.6.01	East China Sea	122.6000	29.7333	-	NMEMC
2000.6.01	East China Sea	122.6000	29.7333	-	NMEMC
2000.6.1	South China Sea	113.9167	22.3667	*Pseudo-nitzschia pungens*	NMEMC
2000.6.1	South China Sea	114.3844	22.4845	*Skeletonema costatum*	NMEMC
2000.6.26	East China Sea	122.9015	30.5900	*Chaetoceros siamense*	NMEMC
2000.7.12	Bohai Sea	121.6000	40.2667	-	NMEMC
2000.7.20	Bohai Sea	117.9333	38.5833	-	NMEMC
2000.7.20	Yellow Sea	120.2500	36.1500	*Noctiluca scintillans*	NMEMC
2000.7.21	Bohai Sea	119.0000	39.2100	-	NMEMC
2000.7.23	Bohai Sea	118.6100	38.6833	-	NMEMC
2000.7.23	Bohai Sea	119.6000	39.8900	-	NMEMC
2000.7.9	Bohai Sea	121.9333	40.3833	-	NMEMC
2000.8.13	Bohai Sea	121.2500	38.5000	-	NMEMC
2000.8.13	Bohai Sea	121.5000	40.0000	-	NMEMC
2000.8.16	Bohai Sea	121.3600	39.7000	-	NMEMC
2000.8.2	Yellow Sea	121.7600	39.0200	-	NMEMC
2000.8.2	Yellow Sea	123.0100	39.5700	-	NMEMC
2000.8.2	Yellow Sea	123.4700	39.7000	-	NMEMC
2000.9.14	Bohai Sea	119.4200	39.6500	-	NMEMC
2000.9.16	East China Sea	119.5333	34.6333	-	NMEMC
2000.9.26	South China Sea	114.3844	22.4845	*Scrippsiella trochoidea*	NMEMC

2000.9.4	East China Sea	118.0500	24.5615	-	NMEMC
2001.1.1-4	South China Sea	114.6917	22.5833	*Noctiluca scintillans*	NMEMC
2001.1.20-23	South China Sea	110.5833	20.8333	-	NMEMC
2001.1.8-12	South China Sea	122.6833	30.8500	*Thalassiosira pacifica*	NMEMC
2001.2.26-3.1	South China Sea	113.4500	22.2500	*Mesodiniu rubrum*	NMEMC
2001.2.6-23	South China Sea	113.7500	22.4167	*Gymnodinium sanguineum*	NMEMC
2001.3	South China Sea	109.4500	18.2333	*Ceratium trichoceros*	NMEMC
2001.3.17-25	South China Sea	113.9167	22.3667	*Thalassiosira pacifica*	NMEMC
2001.3.21	South China Sea	114.1333	22.4000	*Skeletonema costatum*	NMEMC
2001.3.22	South China Sea	114.3000	22.5500	*Gonyaulax polygramma*	NMEMC
2001.3.25	South China Sea	114.2667	22.3667	*Gonyaulax polygramma*	NMEMC
2001.3.5-7	South China Sea	114.5167	22.6667	*Heterosigma akashiwo*	NMEMC
2001.4.12	South China Sea	114.3333	22.4500	*Scrippsiella trochoidea*	NMEMC
2001.4.13	East China Sea	124.5655	29.2700	-	NMEMC
2001.4.15	East China Sea	124.1500	30.6400	-	NMEMC
2001.4.15	East China Sea	124.6100	30.5833	-	NMEMC
2001.4.16	East China Sea	124.3500	30.4500	-	NMEMC
2001.4.18-23	South China Sea	114.3000	22.6167	*Heterosigma akashiwo*	NMEMC
2001.4.2	South China Sea	114.1833	22.3833	*Scrippsiella trochoidea*	NMEMC
2001.4.2	South China Sea	114.2200	22.4667	*Phaeocystis scrobiculata*	NMEMC
2001.4.21	East China Sea	121.0700	27.4900	-	NMEMC
2001.4.23-28	South China Sea	114.7000	22.6667	*Prorocentrum donghaiense*	NMEMC
2001.4.28-5.2	South China Sea	114.3500	22.6000	*Gymnodinium sanguineum*	NMEMC
2001.4.3	South China Sea	113.9500	22.2333	*Scrippsiella trochoidea*	NMEMC
2001.4.4	Yellow Sea	120.3800	36.0600	*Noctiluca scintillans*	NMEMC
2001.4.7	East China Sea	124.5467	29.4000	-	NMEMC
2001.4.9	East China Sea	121.0700	27.4900	-	NMEMC
2001.5.10	East China Sea	122.3333	30.3333	*Prorocentrumdentatum*	NMEMC
2001.5.10	East China Sea	122.4667	31.0115	-	NMEMC

2001.5.10	East China Sea	122.6333	31.3667	*Prorocentrumdentatum*	NMEMC
2001.5.10	East China Sea	122.6667	30.0000	-	NMEMC
2001.5.12	East China Sea	121.9500	30.3000	-	NMEMC
2001.5.12	East China Sea	122.2667	29.7000	*Prorocentrumdentatum*	NMEMC
2001.5.12	East China Sea	122.8600	30.7400	-	NMEMC
2001.5.13-14	South China Sea	115.4167	22.7833	*Gonyaulax spinifera*	NMEMC
2001.5.15	East China Sea	120.3500	26.7000	*Noctiluca scintillans*	NMEMC
2001.5.15	East China Sea	121.0083	27.4500	*Prorocentrumdentatum*	NMEMC
2001.5.17	East China Sea	121.7300	32.0900	-	NMEMC
2001.5.21	East China Sea	121.8100	29.6400	-	NMEMC
2001.5.21	Yellow Sea	123.7500	39.7500	*Noctiluca scintillans*	NMEMC
2001.5.23	East China Sea	120.7500	27.3500	*Prorocentrumdentatum*	NMEMC
2001.5.25	Bohai Sea	118.3900	38.8583	-	NMEMC
2001.5.25	East China Sea	123.7000	33.8667	-	NMEMC
2001.5.29	East China Sea	120.1500	26.7150	-	NMEMC
2001.5.31	Bohai Sea	117.8667	38.9880	*Thalassiosira rotula*	NMEMC
2001.5.31	East China Sea	120.0300	26.5800	-	NMEMC
2001.5.31	East China Sea	120.5400	27.2000	-	NMEMC
2001.6.11-12	Bohai Sea	118.1833	39.2000	*Thalassiosira rotula*	NMEMC
2001.6.15	East China Sea	122.2050	29.5400	-	NMEMC
2001.6.15	East China Sea	122.3150	28.5150	*Skeletonema costatum*	NMEMC
2001.6.16	East China Sea	122.7000	30.3333	*Chaetoceros siamense*	NMEMC
2001.6.16	East China Sea	123.2667	28.5500	-	NMEMC
2001.6.17	East China Sea	113.9000	29.3667	-	NMEMC
2001.6.17	East China Sea	122.2000	29.4300	-	NMEMC
2001.6.17	East China Sea	122.3333	27.7150	*Skeletonema costatum*	NMEMC
2001.6.18	East China Sea	118.0333	24.5333	*Chaetoceros siamense*	NMEMC
2001.6.18	East China Sea	118.0333	24.5333	*Skeletonema costatum*	NMEMC
2001.6.19	Bohai Sea	117.8000	38.6167	-	NMEMC

2001.6.20	East China Sea	121.8333	33.0000	-	NMEMC
2001.6.2-3	Bohai Sea	117.7800	38.9700	*Thalassiosira rotula*	NMEMC
2001.6.27	East China Sea	122.4560	29.4560	-	NMEMC
2001.6.29	East China Sea	123.3667	31.3000	*Skeletonema costatum*	NMEMC
2001.6.8	East China Sea	120.7500	27.3500	-	NMEMC
2001.6.9	East China Sea	120.4150	27.1667	-	NMEMC
2001.7.01	East China Sea	121.9000	28.4556	-	NMEMC
2001.7.01	East China Sea	122.6667	30.7000	-	NMEMC
2001.7.10	Bohai Sea	121.6500	40.1167	-	NMEMC
2001.7.11	South China Sea	114.6800	22.6600	-	NMEMC
2001.7.11	Yellow Sea	120.2600	36.0000	*Mesodiniu rubrum*	NMEMC
2001.7.12	Yellow Sea	120.2800	36.0300	*Mesodiniu rubrum*	NMEMC
2001.7.13	South China Sea	114.6800	22.6600	*Skeletonema costatum*	NMEMC
2001.7.15	Bohai Sea	120.5333	40.5333	*Noctiluca scintillans*	NMEMC
2001.7.17	East China Sea	122.1400	31.3800	-	NMEMC
2001.7.3	East China Sea	118.0500	24.4667	-	NMEMC
2001.7.3	East China Sea	123.1150	31.6333	-	NMEMC
2001.7.7	Bohai Sea	122.1333	40.1167	*Noctiluca scintillans*	NMEMC
2001.8.01	East China Sea	122.8150	30.7000	-	NMEMC
2001.8.12	Bohai Sea	121.8167	40.0500	*Leptocylindrus danicus*	NMEMC
2001.8.12	Bohai Sea	121.8167	40.0500	*Leptocylindrus danicus*	NMEMC
2001.8.19	Bohai Sea	122.0500	40.2900	*Leptocylindrus danicus*	NMEMC
2001.8.24	Yellow Sea	124.1200	39.8100	*Gonyaulax polygramma*	NMEMC
2001.8.25	Bohai Sea	122.0900	40.6300	*Noctiluca scintillans*	NMEMC
2001.8.25	Bohai Sea	122.1000	40.2700	*Mesodiniu rubrum*	NMEMC
2001.8.27	Bohai Sea	122.0800	40.2700	*Mesodiniu rubrum*	NMEMC
2001.9.12	Bohai Sea	122.0800	40.2700	*Mesodiniu rubrum*	NMEMC
2001.9.12	Bohai Sea	122.1000	40.6400	*Noctiluca scintillans*	NMEMC
2001.9.16	Bohai Sea	122.1000	40.2700	-	NMEMC

2001.9.16-20	South China Sea	114.7000	22.6667	*Leptocylindrus danicus*	NMEMC
2001.9.3-6	Bohai Sea	122.0800	40.2700	*Pseudo-nitzschia pungens*	NMEMC
2001.9.3-8	Bohai Sea	122.0800	40.2700	*Chaetoceros socialis*	NMEMC
2001.9.4	Bohai Sea	121.9400	40.8000	-	NMEMC
2001.9.6-12	Bohai Sea	122.1000	40.6400	*Thalassiosira rotula*	NMEMC
2002.1.13-16	South China Sea	117.1667	23.3833	-	NMEMC
2002.10.15	East China Sea	122.3667	29.6667	-	NMEMC
2002.2.6	South China Sea	110.6500	20.9333	-	NMEMC
2002.3.21	South China Sea	114.1500	22.2333	*Noctiluca scintillans*	NMEMC
2002.3.21	South China Sea	114.1500	22.2333	*Noctiluca scintillans*	NMEMC
2002.4.11	East China Sea	122.4167	29.8000	-	NMEMC
2002.4.11	East China Sea	122.7000	30.2167	-	NMEMC
2002.4.13-15	East China Sea	120.3700	26.7033	*Noctiluca scintillans*	NMEMC
2002.4.17	East China Sea	121.0833	27.4667	-	NMEMC
2002.4.17	East China Sea	121.1667	27.6167	-	NMEMC
2002.4.23	East China Sea	120.3700	26.7033	*Noctiluca scintillans*	NMEMC
2002.4.30-5.1	East China Sea	120.0333	26.5000	*Noctiluca scintillans*	NMEMC
2002.5.1	East China Sea	121.2000	27.6167	-	NMEMC
2002.5.10	East China Sea	121.8861	28.4556	-	NMEMC
2002.5.10-11	East China Sea	119.6500	25.5000	*Noctiluca scintillans*	NMEMC
2002.5.11	East China Sea	122.0833	30.0167	-	NMEMC
2002.5.15	East China Sea	122.5833	30.6833	*Prorocentrumdentatum*	NMEMC
2002.5.16	East China Sea	120.0000	26.5833	-	NMEMC
2002.5.16	East China Sea	122.4000	30.0000	-	NMEMC
2002.5.16	East China Sea	122.6667	30.7833	*Prorocentrumdentatum*	NMEMC
2002.5.16	East China Sea	122.7000	30.2333	-	NMEMC
2002.5.16-17	East China Sea	122.8167	30.7333	-	NMEMC
2002.5.16-19	East China Sea	120.2000	26.8167	*Prorocentrumdentatum*	NMEMC
2002.5.17	East China Sea	122.3000	30.2333	*Prorocentrumdentatum*	NMEMC

2002.5.18	East China Sea	121.0833	27.4667	-	NMEMC
2002.5.18	East China Sea	122.4167	29.9000	-	NMEMC
2002.5.18-22	East China Sea	121.5667	28.2833	-	NMEMC
2002.5.19	East China Sea	122.3500	29.6833	-	NMEMC
2002.5.19	East China Sea	122.5167	30.9250	*Mesodiniu rubrum*	NMEMC
2002.5.19	East China Sea	122.6167	30.4833	*Mesodiniu rubrum*	NMEMC
2002.5.23	Bohai Sea	120.0084	40.0417	-	NMEMC
2002.5.2-3	South China Sea	109.1000	21.0333	-	NMEMC
2002.5.26	East China Sea	122.6333	30.8333	-	NMEMC
2002.5.27	East China Sea	120.3700	26.7033	*Noctiluca scintillans*	NMEMC
2002.5.27	East China Sea	122.6567	30.7117	*Mesodiniu rubrum*	NMEMC
2002.5.28	East China Sea	120.1833	26.7833	*Skeletonema costatum*	NMEMC
2002.5.28	East China Sea	122.1667	29.6500	-	NMEMC
2002.5.28	East China Sea	122.2000	29.6167	-	NMEMC
2002.5.28	East China Sea	122.6567	30.7117	-	NMEMC
2002.5.3	East China Sea	122.7000	30.2167	*Noctiluca scintillans*	NMEMC
2002.5.30	South China Sea	114.9667	22.4500	-	NMEMC
2002.5.30	East China Sea	122.8000	30.7117	*Skeletonema costatum*	NMEMC
2002.5.3-7	East China Sea	122.7000	30.2167	*Noctiluca scintillans*	NMEMC
2002.5.4	East China Sea	121.0500	27.4500	-	NMEMC
2002.5.4	East China Sea	121.0833	27.4667	-	NMEMC
2002.5.4-14	East China Sea	120.3000	26.6833	*Prorocentrumdentatum*	NMEMC
2002.5.6	East China Sea	121.8861	28.4556	-	NMEMC
2002.5.6	East China Sea	122.3000	30.2333	*Prorocentrumdentatum*	NMEMC
2002.5.6	East China Sea	122.3333	29.6667	*Prorocentrumdentatum*	NMEMC
2002.5.6	East China Sea	122.4000	29.8000	*Prorocentrum donghaiense*	NMEMC
2002.5.6	East China Sea	122.4000	29.8000	*Prorocentrum donghaiense*	NMEMC
2002.5.6-16	East China Sea	119.8667	26.3000	*Prorocentrumdentatum*	NMEMC
2002.5.7	East China Sea	121.1667	30.1667	*Noctiluca scintillans*	NMEMC

2002.5.7-10	East China Sea	118.0333	24.3833	*Skeletonema costatum*	NMEMC
2002.5.7-9	East China Sea	119.7000	26.3833	*Noctiluca scintillans*	NMEMC
2002.6.11	East China Sea	121.8833	28.4500	-	NMEMC
2002.6.12	East China Sea	121.8000	28.6167	-	NMEMC
2002.6.12	East China Sea	121.8861	28.4556	-	NMEMC
2002.6.12	East China Sea	122.0833	30.0167	-	NMEMC
2002.6.12	East China Sea	123.3584	29.1967	-	NMEMC
2002.6.1-4	East China Sea	120.3700	26.7033	-	NMEMC
2002.6.14-15	Bohai Sea	121.4100	40.5833	*Noctiluca scintillans*	NMEMC
2002.6.16-18	Bohai Sea	119.0227	39.1793	*Noctiluca scintillans*	NMEMC
2002.6.17-7.8	South China Sea	114.5167	22.7167	*Phaeocystis scrobiculata*	NMEMC
2002.6.18	East China Sea	121.8833	28.4500	-	NMEMC
2002.6.19-23	South China Sea	109.1000	21.0333	*Protoperidinium bipes*	NMEMC
2002.6.20	East China Sea	121.1667	30.1667	-	NMEMC
2002.6.20	East China Sea	122.3000	30.2333	*Noctiluca scintillans*	NMEMC
2002.6.21-24	East China Sea	118.0667	24.4667	*Skeletonema costatum*	NMEMC
2002.6.2-3	East China Sea	121.8833	28.4500	-	NMEMC
2002.6.27-29	Bohai Sea	119.0227	39.1793	*Gymnodinium aeruginosum*	NMEMC
2002.6.28-30	Yellow Sea	120.1667	36.0417	*Mesodiniu rubrum*	NMEMC
2002.6.3	Bohai Sea	119.4389	39.5481	*Noctiluca scintillans*	NMEMC
2002.6.3	Bohai Sea	119.5215	39.5500	*Noctiluca scintillans*	NMEMC
2002.6.30	Yellow Sea	121.0578	39.8117	-	NMEMC
2002.6.3-11	East China Sea	119.6500	26.1333	*Pseudo-nitzschia pungens*	NMEMC
2002.6.3-8	East China Sea	118.0917	24.4417	*Skeletonema costatum*	NMEMC
2002.6.4-7	East China Sea	118.2000	24.5667	*Skeletonema costatum*	NMEMC
2002.6.5	South China Sea	113.8417	22.3000	*Skeletonema costatum*	NMEMC
2002.6.6-9	East China Sea	120.3000	26.6833	*Skeletonema costatum*	NMEMC
2002.6.7-8	South China Sea	113.8500	22.3167	-	NMEMC
2002.6.7-8	East China Sea	121.5833	29.5000	*Chaetoceros socialis*	NMEMC

2002.6.7-9	East China Sea	121.5833	29.5000	*Chaetoceros socialis*	NMEMC
2002.7.14-21	Bohai Sea	117.7167	38.9833	*Cochlodinium polykrikoides*	NMEMC
2002.7.15	East China Sea	122.8167	30.7000	-	NMEMC
2002.7.24	East China Sea	120.6306	27.5531	-	NMEMC
2002.7.24	East China Sea	121.5589	28.6317	-	NMEMC
2002.7.25-26	Bohai Sea	119.5833	39.9000	*Chattonella marina*	NMEMC
2002.7.4-5	Bohai Sea	117.7000	38.9833	-	NMEMC
2002.8.10	Bohai Sea	117.8828	38.3083	*Noctiluca scintillans*	NMEMC
2002.8.15	Bohai Sea	117.8828	38.3083	*Skeletonema costatum*	NMEMC
2002.8.19	East China Sea	121.8333	29.5833	*Mesodiniu rubrum*	NMEMC
2002.8.19	East China Sea	122.6567	30.7167	*Skeletonema costatum*	NMEMC
2002.8.19	East China Sea	122.8167	30.7500	-	NMEMC
2002.8.19-21	East China Sea	121.5289	29.5072	*Mesodiniu rubrum*	NMEMC
2002.8.28	East China Sea	122.0833	30.0167	-	NMEMC
2002.9.17-23	East China Sea	121.1000	27.5000	*Gymnodinium sanguineum*	NMEMC
2002.9.3	East China Sea	121.7583	29.6250	*Mesodiniu rubrum*	NMEMC
2002.9.3-5	East China Sea	121.7583	29.5417	*Mesodiniu rubrum*	NMEMC
2002.9.9-10	East China Sea	122.9750	29.8167	-	NMEMC
2003.01.25-28	South China Sea	113.8000	22.5333	-	NMEMC
2003.01.25-30	South China Sea	110.5333	19.9333	-	NMEMC
2003.02.08-11	South China Sea	110.3333	20.4333	-	NMEMC
2003.02.09-12	South China Sea	110.5333	19.9333	-	NMEMC
2003.04.02-07	East China Sea	118.0000	24.7667	-	NMEMC
2003.04.17-19	East China Sea	119.9000	25.4500	-	NMEMC
2003.04.22	East China Sea	122.4833	29.2500	-	NMEMC
2003.04.25	Bohai Sea	119.5833	39.8667	-	NMEMC
2003.04.25	East China Sea	121.0500	27.4667	-	NMEMC
2003.04.25-30	East China Sea	118.1000	24.3333	-	NMEMC
2003.04.26	Bohai Sea	119.6000	39.8056	-	NMEMC

2003.04.26	East China Sea	122.2667	28.7333	-	NMEMC
2003.04.27-29	South China Sea	113.7000	22.1667	-	NMEMC
2003.04.28	East China Sea	121.0833	27.4667	-	NMEMC
2003.04.28	East China Sea	122.4506	31.4167	-	NMEMC
2003.04.28	East China Sea	122.5869	28.7000	-	NMEMC
2003.04.28-29	South China Sea	113.8333	22.4167	-	NMEMC
2003.04.28-30	East China Sea	120.2333	27.2500	-	NMEMC
2003.04.28-30	East China Sea	122.7500	31.2500	-	NMEMC
2003.04.29	East China Sea	122.4533	28.9892	-	NMEMC
2003.05.03-04	East China Sea	120.5667	26.6333	-	NMEMC
2003.05.03-06	East China Sea	119.8333	25.4667	-	NMEMC
2003.05.03-08	East China Sea	120.2333	26.9167	-	NMEMC
2003.05.03-10	Yellow Sea	121.4000	37.6000	*Phaeocystis scrobiculata*	NMEMC
2003.05.04	East China Sea	120.2333	26.9167	-	NMEMC
2003.05.04	East China Sea	120.3667	26.6333	-	NMEMC
2003.05.04	East China Sea	122.5833	29.9167	-	NMEMC
2003.05.04-05	East China Sea	120.0333	26.5167	-	NMEMC
2003.05.05	East China Sea	122.5411	30.6656	-	NMEMC
2003.05.07-09	South China Sea	114.7000	22.5833	-	NMEMC
2003.05.08	East China Sea	122.5833	29.9167	-	NMEMC
2003.05.08-10	East China Sea	121.0833	27.4667	-	NMEMC
2003.05.09	East China Sea	122.6833	29.1342	-	NMEMC
2003.05.09-10	East China Sea	122.8167	30.1214	-	NMEMC
2003.05.10	East China Sea	122.5500	29.4800	-	NMEMC
2003.05.10-15	East China Sea	120.3333	26.8167	-	NMEMC
2003.05.10-17	East China Sea	120.2333	26.9167	-	NMEMC
2003.05.11-12	East China Sea	122.4089	29.2231	-	NMEMC
2003.05.12-14	South China Sea	113.9500	22.5000	-	NMEMC
2003.05.12-14	East China Sea	119.8333	25.4333	-	NMEMC

2003.05.13	East China Sea	119.8000	25.4667	-	NMEMC
2003.05.13-14	East China Sea	122.8167	30.7333	-	NMEMC
2003.05.14	East China Sea	121.8736	28.3631	-	NMEMC
2003.05.14	East China Sea	121.9917	28.3667	-	NMEMC
2003.05.15	East China Sea	121.0567	27.4750	-	NMEMC
2003.05.15	East China Sea	121.9333	29.4167	-	NMEMC
2003.05.18-19	East China Sea	120.0333	26.7167	-	NMEMC
2003.05.19	East China Sea	119.8000	25.4833	-	NMEMC
2003.05.19	East China Sea	122.3667	28.7500	-	NMEMC
2003.05.19	East China Sea	122.4333	30.4500	-	NMEMC
2003.05.19	East China Sea	122.5833	30.9167	-	NMEMC
2003.05.19	East China Sea	122.7992	30.7097	-	NMEMC
2003.05.19-20	East China Sea	120.4333	26.6333	-	NMEMC
2003.05.20-6.23	East China Sea	119.6667	26.2000	-	NMEMC
2003.05.20-24	East China Sea	119.8000	25.4667	-	NMEMC
2003.05.20-28	East China Sea	121.5000	26.7167	-	NMEMC
2003.05.21	East China Sea	122.3500	29.7333	-	NMEMC
2003.05.21	East China Sea	122.5500	30.1000	-	NMEMC
2003.05.21-22	East China Sea	120.1667	26.8000	-	NMEMC
2003.05.22	East China Sea	122.1167	28.8333	-	NMEMC
2003.05.23	East China Sea	122.0967	28.8717	-	NMEMC
2003.05.23	East China Sea	122.2833	29.8000	-	NMEMC
2003.05.23	East China Sea	122.3000	28.7667	-	NMEMC
2003.05.23-24	East China Sea	122.7833	30.7500	-	NMEMC
2003.05.24-06.1	East China Sea	120.2333	26.8000	-	NMEMC
2003.05.24-06.3	East China Sea	120.2167	26.9000	-	NMEMC
2003.05.25	South China Sea	115.8333	22.8167	-	NMEMC
2003.05.25-06.9	East China Sea	121.0833	27.4667	-	NMEMC
2003.05.25-27	East China Sea	120.7000	26.9667	-	NMEMC

2003.05.27	South China Sea	113.9167	22.4833	-	NMEMC
2003.05.28	Bohai Sea	117.7667	40.9500	-	NMEMC
2003.05.28	Bohai Sea	119.9333	40.0000	-	NMEMC
2003.05.28-06.1	East China Sea	120.4333	26.6333	-	NMEMC
2003.05.30	East China Sea	119.7333	26.5833	-	NMEMC
2003.05.30	East China Sea	119.7333	26.5833	-	NMEMC
2003.05.30	East China Sea	120.0333	26.5167	-	NMEMC
2003.05.30	East China Sea	120.1667	26.5000	-	NMEMC
2003.05.30	Bohai Sea	124.1667	39.8333	-	NMEMC
2003.05.30-06.3	Bohai Sea	119.5833	39.6667	-	NMEMC
2003.05.30-06.3	East China Sea	119.9667	26.4667	-	NMEMC
2003.5.30-06.18	East China Sea	119.7333	39.4167	-	NMEMC
2003.05.31	Bohai Sea	118.8667	38.3667	-	NMEMC
2003.05.31	Bohai Sea	120.1000	38.6667	-	NMEMC
2003.06.01	East China Sea	119.9500	26.4833	-	NMEMC
2003.06.01	East China Sea	122.3833	28.7833	-	NMEMC
2003.06.02	Bohai Sea	117.7667	40.9500	-	NMEMC
2003.06.02	Bohai Sea	120.8333	40.3667	-	NMEMC
2003.06.02	East China Sea	122.3167	31.0333	-	NMEMC
2003.06.02-03	Bohai Sea	119.5833	39.8333	-	NMEMC
2003.06.03	East China Sea	117.9000	24.3667	-	NMEMC
2003.06.04	Bohai Sea	118.5333	38.9500	-	NMEMC
2003.06.04	East China Sea	121.8833	29.6000	-	NMEMC
2003.06.04	East China Sea	122.0333	28.8500	-	NMEMC
2003.06.04	East China Sea	122.2333	29.0667	-	NMEMC
2003.06.04-05	East China Sea	117.8667	24.4833	-	NMEMC
2003.06.05	East China Sea	122.0419	31.4008	-	NMEMC
2003.06.05	East China Sea	122.3667	31.1667	-	NMEMC
2003.06.05	East China Sea	122.7667	30.3833	-	NMEMC

2003.06.05	East China Sea	122.9833	29.9667	-	NMEMC
2003.06.05-16	East China Sea	122.5000	27.5500	-	NMEMC
2003.06.06	East China Sea	119.8000	25.4667	-	NMEMC
2003.06.08	East China Sea	122.6833	30.8500	-	NMEMC
2003.06.08	East China Sea	122.8103	30.6481	-	NMEMC
2003.06.09	East China Sea	121.8833	29.6000	-	NMEMC
2003.06.09-10	South China Sea	113.9167	22.4833	-	NMEMC
2003.06.09-10	East China Sea	119.8000	25.4667	-	NMEMC
2003.06.10	East China Sea	121.7833	28.2500	-	NMEMC
2003.06.10-17	East China Sea	120.8167	27.5333	-	NMEMC
2003.06.11-16	East China Sea	121.9667	29.5500	-	NMEMC
2003.06.12	Bohai Sea	119.5833	39.9167	-	NMEMC
2003.06.13	East China Sea	121.1833	27.9500	-	NMEMC
2003.06.13-14	South China Sea	114.5333	22.5833	-	NMEMC
2003.06.14	East China Sea	121.1667	27.9167	-	NMEMC
2003.06.14	East China Sea	122.8000	30.7167	-	NMEMC
2003.06.15-20	South China Sea	114.7333	22.7000	-	NMEMC
2003.06.16	East China Sea	121.1500	27.8833	-	NMEMC
2003.06.16	East China Sea	122.8000	30.6833	-	NMEMC
2003.06.17	East China Sea	121.1500	27.8833	-	NMEMC
2003.06.20	East China Sea	121.9667	30.3000	-	NMEMC
2003.06.21	Bohai Sea	119.4667	39.3333	-	NMEMC
2003.06.22	East China Sea	122.8000	30.1333	-	NMEMC
2003.06.22	East China Sea	122.8333	30.6667	-	NMEMC
2003.06.23	East China Sea	118.1000	24.5500	-	NMEMC
2003.06.23-26	South China Sea	113.9167	22.4833	-	NMEMC
2003.06.23-26	East China Sea	118.1000	24.3333	-	NMEMC
2003.06.24	East China Sea	121.1500	27.8833	-	NMEMC
2003.06.24-25	East China Sea	121.1500	27.8833	-	NMEMC

2003.06.24-25	East China Sea	121.1833	27.9500	-	NMEMC
2003.06.25	East China Sea	122.5000	30.7500	-	NMEMC
2003.06.25-26	East China Sea	118.1667	24.5667	-	NMEMC
2003.06.25-27	Bohai Sea	119.4167	39.7667	-	NMEMC
2003.06.26	East China Sea	120.3833	27.5000	-	NMEMC
2003.06.26	East China Sea	122.2500	30.6667	-	NMEMC
2003.06.28	Yellow Sea	124.1667	39.8333	-	NMEMC
2003.06.28-30	East China Sea	122.3833	31.5000	-	NMEMC
2003.06.28-30	East China Sea	122.4667	31.0000	-	NMEMC
2003.06.28-30	East China Sea	124.3333	31.0000	-	NMEMC
2003.07.01-03	Yellow Sea	121.6833	38.9833	-	NMEMC
2003.07.02-05	East China Sea	118.3333	24.3667	-	NMEMC
2003.07.02-08	Bohai Sea	117.9500	38.9000	-	NMEMC
2003.07.02-08.2	South China Sea	114.6167	22.6333	-	NMEMC
2003.07.03	East China Sea	118.0000	24.3333	-	NMEMC
2003.07.04-11	Yellow Sea	120.4833	35.9500	-	NMEMC
2003.07.06-09	South China Sea	109.0667	21.0167	-	NMEMC
2003.07.07-08	East China Sea	122.7833	30.7500	-	NMEMC
2003.07.14	East China Sea	118.0000	24.3333	-	NMEMC
2003.07.21-23	East China Sea	118.0000	24.3667	-	NMEMC
2003.07.21-24	South China Sea	114.6167	22.6333	-	NMEMC
2003.07.22-26	South China Sea	117.0667	23.5833	-	NMEMC
2003.07.22-28	South China Sea	116.6167	23.1500	-	NMEMC
2003.08.03	South China Sea	116.6167	23.1333	-	NMEMC
2003.08.12-13	Bohai Sea	117.6500	38.5000	-	NMEMC
2003.08.12-15	South China Sea	114.7333	22.8000	-	NMEMC
2003.08.17	East China Sea	122.3667	31.1667	-	NMEMC
2003.08.19-20	Yellow Sea	121.9000	36.7000	-	NMEMC
2003.08.19-22	Yellow Sea	120.3167	36.0500	-	NMEMC

2003.08.24	East China Sea	121.9000	29.5833	-	NMEMC
2003.08.25-27	East China Sea	118.0000	24.3667	-	NMEMC
2003.08.28-30	South China Sea	114.5500	22.7000	-	NMEMC
2003.08.30	East China Sea	122.3167	31.0833	-	NMEMC
2003.10.11-13	East China Sea	119.8000	25.5000	-	NMEMC
2003.11.11	South China Sea	121.1611	27.8694	-	NMEMC
2003.12.31	South China Sea	117.1167	23.2500	-	NMEMC
2004.01.01	South China Sea	117.1000	23.2500	*Phaeocystis scrobiculata*	NMEMC
2004.01.04	South China Sea	113.4500	22.2500	*Mesodiniu rubrum*	NMEMC
2004.01.05	South China Sea	113.4500	22.2500	*Phaeocystis globosa*	NMEMC
2004.01.07	South China Sea	113.2833	22.2500	*Skeletonema costatum*	NMEMC
2004.01.07	South China Sea	113.8167	22.4833	*Skeletonema costatum*	NMEMC
2004.01.07	South China Sea	113.8333	22.4167	*Skeletonema costatum*	NMEMC
2004.01.07	South China Sea	113.9167	22.3667	*Skeletonema costatum*	NMEMC
2004.01.08	South China Sea	113.8333	22.3333	*Skeletonema costatum*	NMEMC
2004.01.08	South China Sea	117.1500	23.3833	*Phaeocystis scrobiculata*	NMEMC
2004.03.22	Yellow Sea	120.2167	36.1333	*Thalassiosira nordenskioeldii*	NMEMC
2004.04.14	South China Sea	110.5833	20.9000	*Mesodiniu rubrum*	NMEMC
2004.04.27	East China Sea	120.4000	26.6000	*Prorocentrumdentatum*	NMEMC
2004.04.29	East China Sea	120.4333	27.1333	*Prorocentrumdentatum*	NMEMC
2004.05.02	East China Sea	120.4333	26.6333	*Prorocentrum donghaiense*	NMEMC
2004.05.02-04	East China Sea	120.4333	27.1333	*Prorocentrumdentatum*	NMEMC
2004.05.05	East China Sea	121.0667	27.4333	*Prorocentrum donghaiense*	NMEMC
2004.05.06	East China Sea	121.0833	27.4667	*Prorocentrum donghaiense*	NMEMC
2004.05.07	East China Sea	122.3000	30.2333	*Prorocentrum donghaiense*	NMEMC
2004.05.08	East China Sea	122.2833	28.8000	*Prorocentrumdentatum*	NMEMC
2004.05.09	East China Sea	120.4333	26.6333	*Prorocentrumdentatum*	NMEMC
2004.05.11	East China Sea	121.0667	27.4333	*Prorocentrumdentatum*	NMEMC
2004.05.12	East China Sea	121.0833	27.4667	*Prorocentrumdentatum*	NMEMC

2004.05.13	East China Sea	122.5333	30.7000	-	NMEMC
2004.05.14	East China Sea	121.0833	27.4667	*Prorocentrumdentatum*	NMEMC
2004.05.14	East China Sea	121.8603	28.4300	*Prorocentrumdentatum*	NMEMC
2004.05.14	East China Sea	122.1575	28.8942	*Prorocentrumdentatum*	NMEMC
2004.05.15	East China Sea	122.3833	30.0000	-	NMEMC
2004.05.16	East China Sea	120.4333	27.1333	*Prorocentrumdentatum*	NMEMC
2004.05.16	East China Sea	122.1575	28.8942	*Prorocentrumdentatum*	NMEMC
2004.05.16	East China Sea	122.3167	29.3500	*Prorocentrumdentatum*	NMEMC
2004.05.17	East China Sea	121.2000	27.6667	*Prorocentrumdentatum*	NMEMC
2004.05.17	East China Sea	122.6333	30.7167	*Prorocentrumdentatum*	NMEMC
2004.05.18	East China Sea	120.8333	26.9833	*Prorocentrumdentatum*	NMEMC
2004.05.18	East China Sea	121.6667	28.4167	*Prorocentrumdentatum*	NMEMC
2004.05.18	East China Sea	121.8717	28.4217	*Prorocentrumdentatum*	NMEMC
2004.05.18	East China Sea	122.5833	29.9000	-	NMEMC
2004.05.18	East China Sea	122.6000	30.2833	-	NMEMC
2004.05.18	East China Sea	122.6333	30.0667	-	NMEMC
2004.05.18	East China Sea	122.7000	30.2167	-	NMEMC
2004.05.18	East China Sea	122.7333	30.7000	*Prorocentrumdentatum*	NMEMC
2004.05.19	East China Sea	121.0000	27.5000	-	NMEMC
2004.05.19	East China Sea	121.1500	27.6167	-	NMEMC
2004.05.19	East China Sea	121.2058	27.8256	*Prorocentrumdentatum*	NMEMC
2004.05.19	East China Sea	121.6167	28.7167	-	NMEMC
2004.05.19	East China Sea	122.4000	29.3833	-	NMEMC
2004.05.20	East China Sea	119.7167	25.6000	*Noctiluca scintillans*	NMEMC
2004.05.20	East China Sea	121.1500	27.8167	*Prorocentrumdentatum*	NMEMC
2004.05.20	East China Sea	121.1500	29.3333	*Prorocentrumdentatum*	NMEMC
2004.05.21	East China Sea	121.1889	27.8692	*Prorocentrumdentatum*	NMEMC
2004.05.21	East China Sea	122.5667	30.0000	-	NMEMC
2004.05.22	East China Sea	121.0833	27.7167	-	NMEMC

2004.05.22	East China Sea	121.2167	28.5000	-	NMEMC
2004.05.22	East China Sea	121.3500	29.7667	-	NMEMC
2004.05.22	East China Sea	121.4500	30.6836	*Prorocentrumdentatum*	NMEMC
2004.05.22	East China Sea	121.5667	29.1500	-	NMEMC
2004.05.22	East China Sea	121.8333	29.5833	-	NMEMC
2004.05.22	East China Sea	122.4000	29.3833	-	NMEMC
2004.05.23	Bohai Sea	120.9003	40.7169	*Noctiluca scintillans*	NMEMC
2004.05.23	Bohai Sea	121.0833	40.7167	*Noctiluca scintillans*	NMEMC
2004.05.23	East China Sea	121.4500	34.7706	*Noctiluca scintillans*	NMEMC
2004.05.24	East China Sea	121.0500	28.8000	-	NMEMC
2004.05.24	East China Sea	121.4333	29.5167	-	NMEMC
2004.05.24	East China Sea	121.9333	29.5000	-	NMEMC
2004.05.24	East China Sea	122.3500	28.9167	-	NMEMC
2004.05.25	East China Sea	119.4833	25.2000	*Noctiluca scintillans*	NMEMC
2004.05.25	East China Sea	122.3667	29.6667	*Prorocentrumdentatum*	NMEMC
2004.05.25	East China Sea	122.4167	29.9000	*Prorocentrumdentatum*	NMEMC
2004.05.25	East China Sea	122.6167	30.0333	-	NMEMC
2004.05.25	East China Sea	122.7833	30.2000	-	NMEMC
2004.05.26	East China Sea	120.6081	27.3783	*Prorocentrumdentatum*	NMEMC
2004.05.26	East China Sea	122.2483	29.2275	*Prorocentrumdentatum*	NMEMC
2004.05.27	East China Sea	120.6833	26.9667	*Noctiluca scintillans*	NMEMC
2004.05.27	East China Sea	121.0692	27.4469	*Noctiluca scintillans*	NMEMC
2004.05.27	East China Sea	121.2236	27.8217	*Prorocentrumdentatum*	NMEMC
2004.05.27	East China Sea	121.8000	28.7000	*Prorocentrumdentatum*	NMEMC
2004.05.28	East China Sea	122.1572	29.0772	*Prorocentrumdentatum*	NMEMC
2004.05.29	East China Sea	121.0667	27.4333	*Prorocentrumdentatum*	NMEMC
2004.05.29	East China Sea	122.7168	30.6836	-	NMEMC
2004.05.31	Bohai Sea	117.7969	38.9483	*Skeletonema costatum*	NMEMC
2004.05.31	East China Sea	118.0667	24.4667	*Chaetoceros siamense*	NMEMC

2004.06.01	East China Sea	122.5000	30.2500	*Skeletonema costatum*	NMEMC
2004.06.03	Bohai Sea	117.6833	38.8500	*Gymnodinium aeruginosum*	NMEMC
2004.06.11	Bohai Sea	117.9097	38.6911	*Rhizosolenia fragilissima*	NMEMC
2004.06.11	Yellow Sea	118.3853	38.4653	-	NMEMC
2004.06.11	East China Sea	122.6833	30.8500	-	NMEMC
2004.06.12	Bohai Sea	117.8333	39.0167	*Heterosigma akashiwo*	NMEMC
2004.06.14	South China Sea	118.0333	24.3833	*Trichodesmium contortum*	NMEMC
2004.06.14	East China Sea	118.0917	24.4872	*Chaetoceros curvisetus*	NMEMC
2004.06.15	East China Sea	110.3667	21.0167	*Chaetoceros curvisetus*	NMEMC
2004.06.15	Bohai Sea	117.6833	38.8500	*Mesodiniu rubrum*	NMEMC
2004.06.15	Bohai Sea	118.0533	38.8033	*Mesodiniu rubrum*	NMEMC
2004.06.15	Yellow Sea	118.8972	38.3572	*Mesodiniu rubrum*	NMEMC
2004.06.15	East China Sea	121.8717	28.4217	*Prorocentrumdentatum*	NMEMC
2004.06.16	South China Sea	114.6333	22.6167	-	NMEMC
2004.06.16	East China Sea	118.0333	24.3833	*Chaetoceros curvisetus*	NMEMC
2004.06.17	Bohai Sea	118.4667	38.2667	*Mesodiniu rubrum*	NMEMC
2004.06.18	Bohai Sea	118.2383	38.3700	*Phaeocystis globosa*	NMEMC
2004.06.19	Bohai Sea	119.5833	39.9000	*Noctiluca scintillans*	NMEMC
2004.06.21	Bohai Sea	117.7067	38.8222	*Mesodiniu rubrum*	NMEMC
2004.06.21	Bohai Sea	117.9667	38.9667	*Mesodiniu rubrum*	NMEMC
2004.06.21	Bohai Sea	118.2164	38.9147	*Mesodiniu rubrum*	NMEMC
2004.06.21	East China Sea	121.0831	27.4608	*Karenia mikimotoi*	NMEMC
2004.06.25	Bohai Sea	119.5000	39.8667	*Noctiluca scintillans*	NMEMC
2004.06.27	East China Sea	118.0667	24.4167	*Chaetoceros curvisetus*	NMEMC
2004.06.28	East China Sea	122.8000	30.7167	*Prorocentrumdentatum*	NMEMC
2004.06.29	South China Sea	109.1333	21.0333	*Phaeocystis scrobiculata*	NMEMC
2004.06.29	East China Sea	121.5167	27.6667	-	NMEMC
2004.06.30	East China Sea	122.7833	30.8000	-	NMEMC
2004.07.05	Bohai Sea	117.7167	38.6222	*Chattonella marina*	NMEMC

2004.07.07	South China Sea	114.5167	22.7167	*Scrippsiella trochoidea*	NMEMC
2004.07.11	East China Sea	122.8000	30.7167	*Skeletonema costatum*	NMEMC
2004.07.12	South China Sea	114.5167	22.7167	*Scrippsiella trochoidea*	NMEMC
2004.07.12	South China Sea	114.7000	22.6667	*Scrippsiella trochoidea*	NMEMC
2004.07.22	East China Sea	122.2500	31.5000	*Skeletonema costatum*	NMEMC
2004.07.28	Bohai Sea	119.1361	38.0953	-	NMEMC
2004.08.02	East China Sea	122.4167	29.8000	-	NMEMC
2004.08.05	South China Sea	114.3333	22.5833	*Protoperidinium bipes*	NMEMC
2004.08.10	South China Sea	114.3000	22.6167	*Protoperidinium bipes*	NMEMC
2004.08.10	Yellow Sea	123.5767	39.5222	-	NMEMC
2004.08.11	Yellow Sea	123.8305	39.7924	*Phaeocystis scrobiculata*	NMEMC
2004.08.12	Yellow Sea	120.3667	36.0833	*Mesodiniu rubrum*	NMEMC
2004.08.16	South China Sea	114.4667	22.5500	*Prorocentrum donghaiense*	NMEMC
2004.08.20	Yellow Sea	121.8500	39.0333	*Noctiluca scintillans*	NMEMC
2004.08.20	East China Sea	122.3667	31.5000	*Skeletonema costatum*	NMEMC
2004.08.26	South China Sea	114.7333	22.7000	-	NMEMC
2004.08.31	South China Sea	110.5833	20.9000	-	NMEMC
2004.09.01	South China Sea	114.2667	22.5833	*Prorocentrum donghaiense*	NMEMC
2004.09.06	South China Sea	114.3000	22.6167	*Prorocentrum donghaiense*	NMEMC
2004.09.08	Bohai Sea	123.4933	39.7200	*Gymnodinium aeruginosum*	NMEMC
2004.09.10	South China Sea	114.6833	22.6667	-	NMEMC
2004.09.18	Bohai Sea	121.4714	37.5117	-	NMEMC
2004.09.25	Yellow Sea	121.8500	39.0333	*Alexandrium tamarense*	NMEMC
2004.09.27	Bohai Sea	123.4933	39.7200	*Gymnodinium sanguineum*	NMEMC
2004.09.29	Yellow Sea	119.4167	34.7167	*Gonyaulax polygramma*	NMEMC
2004.10.05	Yellow Sea	121.6833	38.8667	*Alexandrium tamarense*	NMEMC
2004.10.11	South China Sea	114.7333	22.6000	-	NMEMC
2004.11.10	South China Sea	116.6833	23.3167	*Skeletonema costatum*	NMEMC
2005.01.19-21	South China Sea	110.8333	19.5000	*Trichodesmium contortum*	NMEMC

2005.03.28-4.3	South China Sea	110.4000	21.1167	*Phaeocystis scrobiculata*	NMEMC
2005.04.01	East China Sea	121.7919	27.4433	*Skeletonema costatum*	NMEMC
2005.04.13-21	East China Sea	122.7667	29.8833	*Skeletonema costatum*	NMEMC
2005.04.21	East China Sea	119.8500	26.3500	*Skeletonema costatum*	NMEMC
2005.04.29-30	South China Sea	113.7167	21.9333	*Noctiluca scintillans*	NMEMC
2005.05.01-05	East China Sea	118.0667	24.4000	*Chaetoceros curvisetus*	NMEMC
2005.05.02	East China Sea	121.2842	28.0811	*Noctiluca scintillans*	NMEMC
2005.05.04	East China Sea	122.5633	28.5217	*Noctiluca scintillans*	NMEMC
2005.05.10	East China Sea	122.3667	29.6667	*Prorocentrumdentatum*	NMEMC
2005.05.13	East China Sea	121.0853	27.4328	*Leptocylindrus danicus*	NMEMC
2005.05.14-15	East China Sea	120.1667	26.9000	-	NMEMC
2005.05.16	East China Sea	119.7833	25.4333	*Noctiluca scintillans*	NMEMC
2005.05.17	East China Sea	120.1667	26.8000	*Noctiluca scintillans*	NMEMC
2005.05.18	East China Sea	119.7833	26.2333	*Skeletonema costatum*	NMEMC
2005.05.18	Yellow Sea	121.6775	38.9810	-	NMEMC
2005.05.19-20	Bohai Sea	117.7333	38.6000	-	NMEMC
2005.05.20	Bohai Sea	117.7544	38.3639	-	NMEMC
2005.05.20	Bohai Sea	118.0997	38.1597	-	NMEMC
2005.05.21	South China Sea	108.3283	20.2283	-	NMEMC
2005.05.22	East China Sea	122.4750	29.6567	-	NMEMC
2005.05.24	South China Sea	113.8333	22.1833	*gymnodinium-mikimotoi*	NMEMC
2005.05.24	Bohai Sea	120.1000	37.5486	*Noctiluca scintillans*	NMEMC
2005.05.24	East China Sea	122.8287	30.7250	*Prorocentrumdentatum*	NMEMC
2005.05.25-26	East China Sea	122.3128	29.7535	*Prorocentrumdentatum*	NMEMC
2005.05.26	East China Sea	122.2650	29.7817	*Skeletonema costatum*	NMEMC
2005.05.26-29	East China Sea	122.6833	30.8500	*gymnodinium-mikimotoi*	NMEMC
2005.05.30	East China Sea	120.9867	27.4475	*Prorocentrumdentatum*	NMEMC
2005.05.30	East China Sea	122.7333	30.1333	-	NMEMC
2005.05.31	East China Sea	121.1981	27.8892	*gymnodinium-mikimotoi*	NMEMC

2005.06.01	East China Sea	121.7883	29.5300	*Mesodiniu rubrum*	NMEMC
2005.06.01	East China Sea	121.8800	28.4583	*gymnodinium-mikimotoi*	NMEMC
2005.06.01	East China Sea	122.6683	30.4667	*gymnodinium-mikimotoi*	NMEMC
2005.06.02	Bohai Sea	118.1117	38.7153	*Gymnodinium aeruginosum*	NMEMC
2005.06.02	East China Sea	122.7833	30.9167	*gymnodinium-mikimotoi*	NMEMC
2005.06.03	Bohai Sea	118.5937	38.6506	-	NMEMC
2005.06.03	Bohai Sea	119.3874	38.1898	-	NMEMC
2005.06.03	East China Sea	122.4500	31.5000	*Skeletonema costatum*	NMEMC
2005.06.04	East China Sea	120.1667	26.9000	*gymnodinium-mikimotoi*	NMEMC
2005.06.05	East China Sea	121.2833	28.0931	*gymnodinium-mikimotoi*	NMEMC
2005.06.06	East China Sea	120.7333	26.9167	-	NMEMC
2005.06.06	East China Sea	121.5189	27.2608	*gymnodinium-mikimotoi*	NMEMC
2005.06.06	East China Sea	122.3700	30.1833	*Prorocentrumdentatum*	NMEMC
2005.06.08	East China Sea	122.2333	29.2000	*Prorocentrumdentatum*	NMEMC
2005.06.08	East China Sea	122.2417	28.9833	-	NMEMC
2005.06.08	East China Sea	122.3833	29.3833	-	NMEMC
2005.06.08-09	East China Sea	122.6833	30.7333	*Prorocentrumdentatum*	NMEMC
2005.06.09-13	East China Sea	118.2000	24.6333	*Chaetoceros siamense*	NMEMC
2005.06.10	East China Sea	120.8000	27.5833	*Karenia mikimotoi*	NMEMC
2005.06.10	East China Sea	122.5667	30.7333	*Prorocentrumdentatum*	NMEMC
2005.06.11	East China Sea	121.9167	30.9500	*Prorocentrumdentatum*	NMEMC
2005.06.12-13	East China Sea	121.5167	29.1333	*Prorocentrumdentatum*	NMEMC
2005.06.12-17	East China Sea	118.1500	24.4333	*Chaetoceros curvisetus*	NMEMC
2005.06.13	East China Sea	122.7000	30.5667	-	NMEMC
2005.06.14-15	East China Sea	120.1667	26.9000	*gymnodinium-mikimotoi*	NMEMC
2005.06.14-17	East China Sea	121.0333	27.4667	*Karenia mikimotoi*	NMEMC
2005.06.15	East China Sea	118.0333	24.3833	*Chaetoceros curvisetus*	NMEMC
2005.06.15	East China Sea	121.0681	27.4861	*Karenia mikimotoi*	NMEMC
2005.06.15	East China Sea	121.6167	28.2667	-	NMEMC

2005.06.15-17	East China Sea	119.6667	25.5333	*Noctiluca scintillans*	NMEMC
2005.06.15-21	East China Sea	121.9167	31.5833	*Skeletonema costatum*	NMEMC
2005.06.16	East China Sea	122.2333	30.6167	*Thalassiosira rotula*	NMEMC
2005.06.16	East China Sea	122.8000	30.7167	*Skeletonema costatum*	NMEMC
2005.06.16-18	Bohai Sea	121.5950	40.0440	*Noctiluca scintillans*	NMEMC
2005.06.16-19	East China Sea	122.4167	29.7667	*Prorocentrumdentatum*	NMEMC
2005.06.17	Bohai Sea	119.1000	37.7667	*Phaeocystis scrobiculata*	NMEMC
2005.06.18-21	East China Sea	121.0833	27.4667	*Karenia mikimotoi*	NMEMC
2005.07.04	Bohai Sea	118.9333	37.2000	*Phaeocystis scrobiculata*	NMEMC
2005.07.05	East China Sea	121.8333	29.4500	-	NMEMC
2005.07.05-6	East China Sea	118.1500	24.4333	*Chaetoceros curvisetus*	NMEMC
2005.07.07-11	East China Sea	122.5667	30.7333	*Pseudo-nitzschia pungens*	NMEMC
2005.07.23	East China Sea	121.0667	27.4833	*Karenia mikimotoi*	NMEMC
2005.08.01-5	East China Sea	118.1500	24.4333	*Chaetoceros siamense*	NMEMC
2005.08.23	Bohai Sea	118.9333	37.2833	*Phaeocystis scrobiculata*	NMEMC
2005.08.23	Yellow Sea	121.5125	37.4581	*Leptocylindrus danicus*	NMEMC
2005.08.25	Yellow Sea	123.4500	39.5000	*Thalassiosira rotula*	NMEMC
2005.08.28	Yellow Sea	123.4933	39.7200	-	NMEMC
2005.08.31	East China Sea	118.0333	24.3833	*Skeletonema costatum*	NMEMC
2005.09.10	Yellow Sea	123.5133	39.3550	-	NMEMC
2005.09.12	Yellow Sea	123.4933	39.7200	*Gymnodinium sanguineum*	NMEMC
2005.09.15-18	East China Sea	118.0333	24.3833	*Skeletonema costatum*	NMEMC
2005.09.23-27	Yellow Sea	119.4500	34.7167	*Skeletonema costatum*	NMEMC
2005.09.24-28	Yellow Sea	121.4000	37.5333	*Gymnodinium sanguineum*	NMEMC
2005.09.30-10.3	East China Sea	118.0333	24.3833	*Chaetoceros siamense*	NMEMC
2005.10.06	Yellow Sea	119.4500	34.7167	*Skeletonema costatum*	NMEMC
2005.10.14	South China Sea	113.2500	21.9167	*Gymnodinium sanguineum*	NMEMC
2005.10.19-20	South China Sea	114.7000	22.6667	*Ceratium trichoceros*	NMEMC
2005.10.21	Yellow Sea	119.4500	34.7167	*Gymnodinium geminatum*	NMEMC

2005.10.29	Yellow Sea	119.4500	34.7167	*Gymnodinium geminatum*	NMEMC
2005.11.22	South China Sea	115.3667	22.7167	*Phaeocystis scrobiculata*	NMEMC
2005.12.04	South China Sea	116.8333	23.3167	*Phaeocystis scrobiculata*	NMEMC
2006.01.06	East China Sea	121.5333	29.4500	-	NMEMC
2006.02.09	South China Sea	113.5882	22.2548	*Phaeocystis globosa*	NMEMC
2006.02.21	South China Sea	113.9000	29.3667	*Phaeocystis globosa*	NMEMC
2006.04.19	East China Sea	122.6333	28.9000	*Noctiluca scintillans*	NMEMC
2006.04.19	East China Sea	123.4500	29.7833	-	NMEMC
2006.04.24-29	South China Sea	110.4333	20.0667	*Cochlodinium polykrikoides*	NMEMC
2006.04.27	South China Sea	113.8003	22.1500	*Cochlodinium polykrikoides*	NMEMC
2006.04.28	South China Sea	109.5500	18.2500	*Skeletonema costatum*	NMEMC
2006.04.29	East China Sea	124.9502	28.9501	-	NMEMC
2006.04.7-8	East China Sea	124.9500	28.8833	-	NMEMC
2006.05.03	East China Sea	122.3167	28.7833	-	NMEMC
2006.05.03	East China Sea	122.6167	30.0833	-	NMEMC
2006.05.06	East China Sea	121.0500	27.4500	*Pseudo-nitzschia pungens*	NMEMC
2006.05.08	East China Sea	121.9333	29.5833	*Skeletonema costatum*	NMEMC
2006.05.08	Yellow Sea	123.0667	39.3833	*Noctiluca scintillans*	NMEMC
2006.05.09	East China Sea	119.7667	25.5333	*Noctiluca scintillans*	NMEMC
2006.05.09	East China Sea	121.5736	29.5117	*Skeletonema costatum*	NMEMC
2006.05.09	East China Sea	122.4002	29.9002	*Prorocentrumdentatum*	NMEMC
2006.05.09-12	South China Sea	110.4333	21.1333	*Skeletonema costatum*	NMEMC
2006.05.11	Bohai Sea	120.3501	39.4336	-	NMEMC
2006.05.11	East China Sea	122.5167	29.6833	-	NMEMC
2006.05.11	East China Sea	122.7335	30.7000	*Prorocentrumdentatum*	NMEMC
2006.05.14	East China Sea	122.7167	31.0000	*Prorocentrumdentatum*	NMEMC
2006.05.20	East China Sea	120.3667	26.7001	-	NMEMC
2006.05.20	East China Sea	122.3000	29.0000	*Prorocentrum donghaiense*	NMEMC
2006.05.22	East China Sea	120.2000	26.7500	-	NMEMC

2006.05.22	East China Sea	121.1200	27.4783	*Prorocentrumdentatum*	NMEMC
2006.05.22	East China Sea	121.8786	28.4536	*Prorocentrumdentatum*	NMEMC
2006.05.31	East China Sea	121.0533	27.4656	*Prorocentrumdentatum*	NMEMC
2006.06.01	East China Sea	120.1833	26.7833	-	NMEMC
2006.06.01	East China Sea	120.2333	26.9000	*Prorocentrumdentatum*	NMEMC
2006.06.01	East China Sea	120.3667	26.7000	-	NMEMC
2006.06.02	Bohai Sea	119.4956	39.8069	-	NMEMC
2006.06.02	Bohai Sea	119.6458	39.8964	-	NMEMC
2006.06.02	Bohai Sea	119.6458	39.8964	-	NMEMC
2006.06.04	Bohai Sea	117.7731	38.7578	-	NMEMC
2006.06.04	Bohai Sea	118.7514	39.0683	-	NMEMC
2006.06.04	Bohai Sea	119.0550	39.1500	-	NMEMC
2006.06.06	East China Sea	119.8667	25.4833	*Prorocentrum donghaiense*	NMEMC
2006.06.06	East China Sea	120.1333	26.6833	-	NMEMC
2006.06.08	East China Sea	120.1000	26.7167	*Prorocentrumdentatum*	NMEMC
2006.06.08	East China Sea	122.4164	29.9167	*Karenia mikimotoi*	NMEMC
2006.06.10-11	East China Sea	121.0597	27.4772	*Noctiluca scintillans*	NMEMC
2006.06.11	Bohai Sea	117.8039	38.6242	-	NMEMC
2006.06.11	Bohai Sea	118.6000	38.3139	-	NMEMC
2006.06.11	East China Sea	122.4000	29.8833	-	NMEMC
2006.06.11	East China Sea	122.4667	29.9002	-	NMEMC
2006.06.12	East China Sea	118.6667	24.6167	-	NMEMC
2006.06.12	East China Sea	118.9167	24.8500	-	NMEMC
2006.06.12	East China Sea	119.5500	25.1333	-	NMEMC
2006.06.12	East China Sea	119.7833	25.4667	-	NMEMC
2006.06.12	East China Sea	122.2000	27.6167	-	NMEMC
2006.06.12	East China Sea	122.2167	29.0833	-	NMEMC
2006.06.13	East China Sea	122.1000	28.9500	*Karenia mikimotoi*	NMEMC
2006.06.14	East China Sea	118.7833	24.8000	-	NMEMC

2006.06.15	East China Sea	122.1668	29.1334	*Karenia mikimotoi*	NMEMC
2006.06.15	East China Sea	122.6001	30.8167	*Prorocentrumdentatum*	NMEMC
2006.06.16	East China Sea	121.9019	28.4650	*Karenia mikimotoi*	NMEMC
2006.06.17	East China Sea	120.9806	27.4597	*Thalassiosira pacifica*	NMEMC
2006.06.18	East China Sea	122.6167	30.1167	-	NMEMC
2006.06.20	East China Sea	121.4703	28.2031	*Karenia mikimotoi*	NMEMC
2006.06.20	East China Sea	122.4111	30.2158	*Prorocentrum donghaiense*	NMEMC
2006.06.21	East China Sea	117.4000	23.7000	*Asterionella japonica*	NMEMC
2006.06.21	East China Sea	120.4303	27.1647	*Karenia mikimotoi*	NMEMC
2006.06.21	East China Sea	121.1976	27.8607	*Skeletonema costatum*	NMEMC
2006.06.21	East China Sea	122.4167	29.9000	*Prorocentrumdentatum*	NMEMC
2006.06.21-22	East China Sea	118.0667	24.4668	*Skeletonema costatum*	NMEMC
2006.06.24	East China Sea	121.6167	29.5333	-	NMEMC
2006.06.24	East China Sea	122.2833	29.3833	-	NMEMC
2006.06.24	East China Sea	122.2833	29.7333	-	NMEMC
2006.06.25	East China Sea	118.2075	24.3833	*Chaetoceros socialis*	NMEMC
2006.06.26	Bohai Sea	117.7833	38.8500	*Heterosigma akashiwo*	NMEMC
2006.06.26	East China Sea	118.1167	24.5000	*Skeletonema costatum*	NMEMC
2006.07.05	East China Sea	118.0667	24.4667	*Chaetoceros curvisetus*	NMEMC
2006.07.06	East China Sea	121.1536	27.8610	*Thalassiosira nordenskioeldii*	NMEMC
2006.07.06-11	South China Sea	109.9667	18.1667	*Dactyliosolen fragilissimus*	NMEMC
2006.07.06-12	South China Sea	109.4500	18.2333	*Gonyaulax polygramma*	NMEMC
2006.07.19-08.3	South China Sea	114.5167	22.7000	*Scrippsiella trochoidea*	NMEMC
2006.07.21	East China Sea	118.1925	24.5261	*Chaetoceros siamense*	NMEMC
2006.07.21	East China Sea	122.6050	30.7003	*Skeletonema costatum*	NMEMC
2006.07.23	East China Sea	118.1500	24.4333	*Chaetoceros siamense*	NMEMC
2006.07.28-30	South China Sea	116.7000	23.3333	*Skeletonema costatum*	NMEMC
2006.07.29	East China Sea	122.5000	30.0000	-	NMEMC
2006.07.31	East China Sea	121.5833	29.4833	*Mesodiniu rubrum*	NMEMC

2006.07.31	East China Sea	123.0000	32.0000	-	NMEMC
2006.08.08-11	South China Sea	114.5167	22.7167	*Scrippsiella trochoidea*	NMEMC
2006.08.08-11	Bohai Sea	117.8167	38.9333	*Noctiluca scintillans*	NMEMC
2006.08.27	East China Sea	121.1200	27.4783	*Nitzschia delicatissima*	NMEMC
2006.09.05	East China Sea	118.0333	24.3833	*Skeletonema costatum*	NMEMC
2006.09.14-21	Bohai Sea	120.9000	38.2833	*Alexandrium catenella*	NMEMC
2006.10.02	Yellow Sea	119.2333	34.8333	*Gymnodinium geminatum*	NMEMC
2006.10.08-19	Bohai Sea	117.8333	38.8567	*Phaeocystis globosa*	NMEMC
2006.10.18	South China Sea	110.4333	21.1333	*Skeletonema costatum*	NMEMC
2006.10.22-11.5	Bohai Sea	117.7833	38.4000	*Phaeocystis globosa*	NMEMC
2006.10.22-26	South China Sea	113.5667	22.2833	*Thalassiosira nordenskioeldii*	NMEMC
2006.10.23	South China Sea	113.6167	22.4167	*Cochlodinium polykrikoides*	NMEMC
2006.10.25	South China Sea	113.5833	22.2500	*Thalassiosira nordenskioeldii*	NMEMC
2006.11.03	East China Sea	123.1306	30.8356	*Noctiluca scintillans*	NMEMC
2006.11.18	South China Sea	113.5833	22.2833	*Cochlodinium polykrikoides*	NMEMC
2006.12.03	South China Sea	115.4167	22.7833	-	NMEMC
2006.3.11	East China Sea	117.5333	23.6667	*Thalassiosira pacifica*	NMEMC
2007.01.12	East China Sea	118.1667	24.5000	*Skeletonema costatum*	NMEMC
2007.01.26	South China Sea	114.2500	22.5833	*Phaeocystis scrobiculata*	NMEMC
2007.01.31	South China Sea	116.7500	23.3333	*Phaeocystis globosa*	NMEMC
2007.02.06	South China Sea	117.0167	23.4168	*Phaeocystis globosa*	NMEMC
2007.02.08	South China Sea	113.5931	22.2548	*Phaeocystis globosa*	NMEMC
2007.02.12	East China Sea	117.5333	23.8333	*Thalassiosira pacifica*	NMEMC
2007.03.25	South China Sea	111.9539	21.6478	*Akashiwo sanguinea*	NMEMC
2007.04.09	East China Sea	120.5000	29.8833	-	NMEMC
2007.04.09	East China Sea	122.8333	30.5000	-	NMEMC
2007.04.10	East China Sea	122.6667	30.1333	-	NMEMC
2007.04.11	East China Sea	122.4333	29.3833	-	NMEMC
2007.05.03	East China Sea	118.2128	24.5278	*Chaetoceros compressus*	NMEMC

2007.05.03	East China Sea	122.4333	28.9500	-	NMEMC
2007.05.05	Bohai Sea	117.8372	38.8411	*Skeletonema costatum*	NMEMC
2007.05.05	East China Sea	121.0639	27.4642	*Skeletonema costatum*	NMEMC
2007.05.05	East China Sea	122.3972	31.6000	*Skeletonema costatum*	NMEMC
2007.05.05	East China Sea	123.0335	29.2501	-	NMEMC
2007.05.09	East China Sea	120.1667	26.8583	*Skeletonema costatum*	NMEMC
2007.05.09	East China Sea	121.8750	28.4208	*Skeletonema costatum*	NMEMC
2007.05.12	East China Sea	119.9061	26.3553	*Scrippsiella trochoidea*	NMEMC
2007.05.23	East China Sea	119.9028	26.3606	*Noctiluca scintillans*	NMEMC
2007.05.23	East China Sea	121.8453	28.4719	*Prorocentrumdentatum*	NMEMC
2007.05.26	East China Sea	121.0579	27.4788	*Prorocentrumdentatum*	NMEMC
2007.05.27	South China Sea	114.3058	22.5967	*Protoperidinium bipes*	NMEMC
2007.05.27	East China Sea	121.0674	27.4486	*Prorocentrumdentatum*	NMEMC
2007.05.28	East China Sea	118.0647	24.4689	*Chaetoceros siamense*	NMEMC
2007.05.29	East China Sea	118.2097	24.4944	*Chaetoceros curvisetus*	NMEMC
2007.05.29	East China Sea	122.2335	29.2502	-	NMEMC
2007.05.30	East China Sea	121.0346	27.4473	*Prorocentrumdentatum*	NMEMC
2007.05.30	East China Sea	121.6036	28.2558	*Prorocentrumdentatum*	NMEMC
2007.05.31	South China Sea	113.8167	22.1500	*Cochlodinium polykrikoides*	NMEMC
2007.05.31	East China Sea	122.8333	29.9167	-	NMEMC
2007.06.05	South China Sea	113.9456	22.4892	*Cochlodinium polykrikoides*	NMEMC
2007.06.05	East China Sea	120.1033	26.7494	*Prorocentrumdentatum*	NMEMC
2007.06.06	East China Sea	120.2606	26.8614	*Noctiluca scintillans*	NMEMC
2007.06.07	Yellow Sea	120.3183	36.0608	*Heterosigma akashiwo*	NMEMC
2007.06.07	East China Sea	122.1064	29.7211	*Navicula tenera*	NMEMC
2007.06.09	East China Sea	119.8833	26.3333	*Karenia mikimotoi*	NMEMC
2007.06.11	East China Sea	119.7667	25.4500	*Karenia mikimotoi*	NMEMC
2007.06.12	East China Sea	119.7667	25.5333	*Karenia mikimotoi*	NMEMC
2007.06.13	East China Sea	119.5000	25.6333	*Pseudo-nitzschia pungens*	NMEMC

2007.06.17	East China Sea	121.0054	27.0167	*Karenia mikimotoi*	NMEMC
2007.06.20	East China Sea	120.5169	27.2528	*Karenia mikimotoi*	NMEMC
2007.06.21	East China Sea	118.0672	24.5114	*Skeletonema costatum*	NMEMC
2007.06.22	East China Sea	118.2128	24.5278	*Chaetoceros siamense*	NMEMC
2007.06.22	East China Sea	121.8500	28.4792	*Karenia mikimotoi*	NMEMC
2007.06.23	East China Sea	122.6500	30.6833	-	NMEMC
2007.06.27	East China Sea	122.4500	29.5000	-	NMEMC
2007.07.01	East China Sea	122.6833	30.8500	-	NMEMC
2007.07.02	East China Sea	122.3833	28.9167	-	NMEMC
2007.07.03	East China Sea	118.0492	24.4428	*Skeletonema costatum*	NMEMC
2007.07.06	East China Sea	122.4100	29.9186	*Scrippsiella trochoidea*	NMEMC
2007.07.10	Yellow Sea	119.5000	34.8000	*Thalassiosira pacifica*	NMEMC
2007.07.10	East China Sea	122.5833	30.7000	*Skeletonema costatum*	NMEMC
2007.07.11	East China Sea	122.5300	30.1908	*Skeletonema costatum*	NMEMC
2007.07.13	Bohai Sea	119.5278	39.8675	*Heterosigma akashiwo*	NMEMC
2007.07.17	East China Sea	122.5000	29.4000	*Chaetoceros curvisetus*	NMEMC
2007.07.18	Yellow Sea	119.4333	34.7667	*Thalassiosira pacifica*	NMEMC
2007.07.20	East China Sea	122.3834	30.2002	*Chaetoceros compressus*	NMEMC
2007.07.21	East China Sea	122.2333	28.9333	*Gonyaulax spinifera*	NMEMC
2007.07.21	East China Sea	122.2833	29.1000	*Gonyaulax spinifera*	NMEMC
2007.07.22	East China Sea	121.8335	27.8333	*Skeletonema costatum*	NMEMC
2007.07.22	East China Sea	121.8881	28.4789	*Gonyaulax polygramma*	NMEMC
2007.07.22	East China Sea	122.5200	29.8789	*Chaetoceros compressus*	NMEMC
2007.07.23	East China Sea	122.6139	29.9069	*Chaetoceros compressus*	NMEMC
2007.07.25	East China Sea	121.8392	29.6850	*Karenia mikimotoi*	NMEMC
2007.07.26	East China Sea	119.6553	22.6383	*Chaetoceros curvisetus*	NMEMC
2007.07.27	East China Sea	122.8001	30.4333	-	NMEMC
2007.07.28	South China Sea	116.7506	23.3314	-	NMEMC
2007.07.29	East China Sea	122.4058	29.9528	-	NMEMC

2007.07.31	East China Sea	122.4502	30.2000	*Chaetoceros compressus*	NMEMC
2007.08.02	East China Sea	122.3928	29.8597	*Leptocylindrus danicus*	NMEMC
2007.08.02	East China Sea	122.7167	30.2167	-	NMEMC
2007.08.02	East China Sea	122.7833	30.6667	-	NMEMC
2007.08.06	East China Sea	118.6533	24.6325	*Skeletonema costatum*	NMEMC
2007.08.16	East China Sea	122.5667	29.8833	-	NMEMC
2007.08.21	Bohai Sea	120.2156	40.0411	*Gymnodinium geminatum*	NMEMC
2007.08.23	Bohai Sea	119.8335	39.9168	*Gymnodinium geminatum*	NMEMC
2007.08.23	East China Sea	121.7428	29.5394	*Skeletonema costatum*	NMEMC
2007.08.24	Bohai Sea	119.4733	39.8142	*Rhizosolenia fragilissima*	NMEMC
2007.08.24	East China Sea	119.7833	26.4000	*Skeletonema costatum*	NMEMC
2007.08.24	East China Sea	121.1688	27.9760	*Skeletonema costatum*	NMEMC
2007.08.25	Bohai Sea	119.5642	39.9061	*gymnodinium-mikimotoi*	NMEMC
2007.08.27	East China Sea	121.2833	29.2000	-	NMEMC
2007.08.27	East China Sea	121.5667	28.8500	-	NMEMC
2007.08.27	East China Sea	122.5333	29.5000	-	NMEMC
2007.08.30	Yellow Sea	121.4433	37.5081	*Gymnodinium sanguineum*	NMEMC
2007.09.08	South China Sea	115.3508	22.7731	*Phaeocystis globosa*	NMEMC
2007.09.16	South China Sea	116.7506	23.3314	*Phaeocystis scrobiculata*	NMEMC
2007.09.25	Yellow Sea	120.5400	36.1106	*Gonyaulax spinifera*	NMEMC
2007.09.27	East China Sea	122.3242	29.7711	-	NMEMC
2007.09.29	East China Sea	122.1833	29.3333	-	NMEMC
2007.09.29	East China Sea	122.5500	29.3333	*Skeletonema costatum*	NMEMC
2007.09.29	East China Sea	122.6667	30.4667	-	NMEMC
2007.10.16	Bohai Sea	117.7717	39.0894	*Phaeocystis globosa*	NMEMC
2007.11.10	Bohai Sea	117.7717	39.1728	*Gonyaulax polygramma*	NMEMC
2007.11.12	Bohai Sea	117.7717	39.1394	*Gonyaulax polygramma*	NMEMC
2008.01.08	South China Sea	114.3992	22.6133	-	NMEMC
2008.02.19	South China Sea	113.9722	22.5217	*Heterosigma akashiwo*	NMEMC

2008.02.27	Yellow Sea	121.7408	38.9678	*Thalassiosira nordenskioeldii*	NMEMC
2008.03.10	East China Sea	119.8500	26.3500	-	NMEMC
2008.03.12	East China Sea	122.9175	30.5667	-	NMEMC
2008.03.13	South China Sea	114.1397	22.5531	*Noctiluca scintillans*	NMEMC
2008.03.16	East China Sea	114.1397	22.5625	*Akashiwo sanguinea*	NMEMC
2008.03.17	East China Sea	120.0383	27.4433	*Thalassiosira nordenskioeldii*	NMEMC
2008.03.19	South China Sea	114.4861	22.5358	*Noctiluca scintillans*	NMEMC
2008.03.21	East China Sea	122.7001	29.8835	-	NMEMC
2008.03.26	East China Sea	121.5122	29.5061	*Skeletonema costatum*	NMEMC
2008.04.22	South China Sea	114.3236	22.6031	*Gonyaulax polygramma*	NMEMC
2008.04.24	East China Sea	122.7711	31.0611	-	NMEMC
2008.04.28	East China Sea	119.8839	26.3083	*Prorocentrum donghaiense*	NMEMC
2008.04.29	East China Sea	119.8044	25.5064	*Prorocentrum donghaiense*	NMEMC
2008.04.30	East China Sea	119.8000	26.2500	*Prorocentrum donghaiense*	NMEMC
2008.05.01	East China Sea	120.3328	26.7061	*Prorocentrumdentatum*	NMEMC
2008.05.01	East China Sea	120.5871	27.3848	*Karenia mikimotoi*	NMEMC
2008.05.02	East China Sea	121.0750	27.4836	*Prorocentrumdentatum*	NMEMC
2008.05.03	East China Sea	121.2200	27.8239	*Karenia mikimotoi*	NMEMC
2008.05.03	East China Sea	122.2722	29.5347	-	NMEMC
2008.05.03	East China Sea	122.3167	29.1667	-	NMEMC
2008.05.05	East China Sea	122.2733	28.8867	-	NMEMC
2008.05.05	East China Sea	122.4400	29.8844	*Prorocentrumdentatum*	NMEMC
2008.05.06	East China Sea	120.5383	27.4133	*Prorocentrumdentatum*	NMEMC
2008.05.06	East China Sea	122.7089	30.1958	-	NMEMC
2008.05.07	East China Sea	120.4000	26.8556	*Prorocentrumdentatum*	NMEMC
2008.05.07	East China Sea	122.6833	30.5000	-	NMEMC
2008.05.08	East China Sea	120.1053	26.8803	*Prorocentrumdentatum*	NMEMC
2008.05.08	East China Sea	120.2834	26.6667	*Prorocentrumdentatum*	NMEMC
2008.05.11	East China Sea	122.8247	30.6956	*Prorocentrumdentatum*	NMEMC

2008.05.12	East China Sea	119.8667	26.3569	*Prorocentrum donghaiense*	NMEMC
2008.05.12	East China Sea	120.5442	27.3392	*Prorocentrumdentatum*	NMEMC
2008.05.14	East China Sea	122.5389	29.8564	-	NMEMC
2008.05.15	East China Sea	121.8611	28.4628	*Prorocentrumdentatum*	NMEMC
2008.05.16	East China Sea	121.0578	27.4786	*Prorocentrumdentatum*	NMEMC
2008.05.16	East China Sea	122.6500	30.7667	-	NMEMC
2008.05.18	East China Sea	121.8286	29.6156	-	NMEMC
2008.05.20	Bohai Sea	119.4667	34.8167	*Heterosigma akashiwo*	NMEMC
2008.05.20	East China Sea	121.6500	28.5167	-	NMEMC
2008.05.20	East China Sea	122.4092	29.8208	-	NMEMC
2008.05.20	East China Sea	122.5053	29.5206	-	NMEMC
2008.05.21	East China Sea	122.8667	30.7833	-	NMEMC
2008.05.22	East China Sea	122.0383	28.7167	-	NMEMC
2008.05.23	East China Sea	121.5833	27.4167	*Noctiluca scintillans*	NMEMC
2008.05.23	East China Sea	122.1500	29.0500	*Prorocentrumdentatum*	NMEMC
2008.05.27	East China Sea	122.2964	29.8158	-	NMEMC
2008.05.31	East China Sea	122.4556	30.4944	*Prorocentrumdentatum*	NMEMC
2008.06.02	East China Sea	122.5886	30.6483	-	NMEMC
2008.06.03	East China Sea	122.4964	30.1450	*Phaeocystis scrobiculata*	NMEMC
2008.06.10	East China Sea	120.0989	26.7583	*Pseudo-nitzschia pungens*	NMEMC
2008.07.15	East China Sea	118.1742	24.5878	*Chaetoceros siamense*	NMEMC
2008.07.16	East China Sea	122.4550	30.1533	*Ceratium trichoceros*	NMEMC
2008.08.05	East China Sea	122.8667	30.1000	-	NMEMC
2008.08.05	East China Sea	123.5000	31.8333	*Skeletonema costatum*	NMEMC
2009.01.7	East China Sea	121.5122	29.5061	*Skeletonema costatum*	NMEMC
2009.02.05	East China Sea	118.0750	24.4250	*Akashiwo sanguinea*	NMEMC
2009.02.14	South China Sea	114.4094	22.6094	*Noctiluca scintillans*	NMEMC
2009.02.16-22	South China Sea	116.9667	23.2000	*Thalassiosira pacifica*	NMEMC
2009.02.24	South China Sea	114.4350	22.5750	*Noctiluca scintillans*	NMEMC

2009.04.03	Yellow Sea	120.3803	36.0603	*Noctiluca scintillans*	NMEMC
2009.04.09	East China Sea	122.8750	31.2500	-	NMEMC
2009.04.09-17	Yellow Sea	121.5681	36.7194	*Noctiluca scintillans*	NMEMC
2009.04.17	Yellow Sea	121.2539	37.5744	*Noctiluca scintillans*	NMEMC
2009.04.22	Yellow Sea	119.5167	34.8167	-	NMEMC
2009.04.23	East China Sea	120.5906	27.3514	*Mesodiniu rubrum*	NMEMC
2009.04.28	East China Sea	121.8773	27.5500	*Gymnodinium aeruginosum*	NMEMC
2009.04.29	East China Sea	121.0608	27.4497	-	NMEMC
2009.05.02	East China Sea	121.6117	28.2531	*Mesodiniu rubrum*	NMEMC
2009.05.02	East China Sea	121.8917	28.4333	*Prorocentrum donghaiense*	NMEMC
2009.05.02	East China Sea	122.3000	28.6500	-	NMEMC
2009.05.04	East China Sea	121.1702	27.8196	*Karenia mikimotoi*	NMEMC
2009.05.05	East China Sea	119.8450	26.3344	*Skeletonema costatum*	NMEMC
2009.05.05-07	East China Sea	121.6667	28.2553	*Karenia mikimotoi*	NMEMC
2009.05.06	East China Sea	122.4707	31.6968	*Skeletonema costatum*	NMEMC
2009.05.07-12	Yellow Sea	119.5636	35.3933	*Noctiluca scintillans*	NMEMC
2009.05.07-12	East China Sea	120.5345	27.3399	*Prorocentrum donghaiense*	NMEMC
2009.05.08-10	East China Sea	120.1638	26.9259	*Prorocentrum donghaiense*	NMEMC
2009.05.09	East China Sea	124.0167	28.6833	-	NMEMC
2009.05.14-20	East China Sea	122.8333	30.7083	*Prorocentrum donghaiense*	NMEMC
2009.05.15-16	East China Sea	121.6267	28.2583	*Noctiluca scintillans*	NMEMC
2009.05.16	East China Sea	118.2931	24.1269	*Noctiluca scintillans*	NMEMC
2009.05.17	East China Sea	122.7500	31.5417	*Prorocentrum donghaiense*	NMEMC
2009.05.17-24	East China Sea	119.4336	25.2089	*Noctiluca scintillans*	NMEMC
2009.05.19	East China Sea	122.5167	30.7667	*Prorocentrum donghaiense*	NMEMC
2009.05.19	East China Sea	122.5833	30.0667	-	NMEMC
2009.05.19-30	East China Sea	123.5500	30.4667	-	NMEMC
2009.05.23-24	East China Sea	119.8072	25.5164	*Noctiluca scintillans*	NMEMC
2009.05.26	Bohai Sea	119.2903	39.7458	*Noctiluca scintillans*	NMEMC

2009.05.26	Yellow Sea	121.3417	36.6750	*Noctiluca scintillans*	NMEMC
2009.05.26-30	Bohai Sea	117.9569	39.0819	*Thalassiosira nordenskioeldii*	NMEMC
2009.05.30	East China Sea	120.2598	26.9396	*Nitzschia closterium*	NMEMC
2009.05.30	East China Sea	120.3678	26.6950	*Noctiluca scintillans*	NMEMC
2009.05.31	Bohai Sea	118.2136	38.7433	-	NMEMC
2009.05.31-6.13	Bohai Sea	117.8161	38.8531	*Heterosigma akashiwo*	NMEMC
2009.06.01-03	East China Sea	120.5751	27.3960	*Karenia mikimotoi*	NMEMC
2009.06.02-04	East China Sea	121.0581	27.4789	*Heterosigma akashiwo*	NMEMC
2009.06.04	East China Sea	122.8333	30.3250	-	NMEMC
2009.06.11	East China Sea	123.7917	31.7750	-	NMEMC
2009.06.13	East China Sea	122.8667	29.6500	-	NMEMC
2009.06.13	East China Sea	123.1667	29.3333	-	NMEMC
2009.06.16-20	East China Sea	121.0113	28.3667	*Karenia mikimotoi*	NMEMC
2009.06.17	East China Sea	124.6333	31.9500	-	NMEMC
2009.06.17-22	East China Sea	122.6583	29.8833	*Skeletonema costatum*	NMEMC
2009.06.17-22	East China Sea	122.7167	30.5750	*Skeletonema costatum*	NMEMC
2009.06.18-20	East China Sea	121.1600	27.8669	*Karenia mikimotoi*	NMEMC
2009.06.22	East China Sea	120.4000	34.5333	-	NMEMC
2009.06.23-29	East China Sea	121.6839	28.2714	*Karenia mikimotoi*	NMEMC
2009.07.18	Yellow Sea	119.3833	34.7750	*Chaetoceros socialis*	NMEMC
2009.07.27	East China Sea	122.6583	29.9000	-	NMEMC
2009.07.28	Yellow Sea	121.7727	36.8730	*Chattonella marina*	NMEMC
2009.7.31-8.4	East China Sea	118.1833	24.5356	*Skeletonema costatum*	NMEMC
2009.08.01	Bohai Sea	117.9167	39.0583	*Skeletonema costatum*	NMEMC
2009.08.04-05	East China Sea	122.5083	29.8833	*Chaetoceros socialis*	NMEMC
2009.08.04-06	East China Sea	122.6583	29.9000	*Rhizosolenia fragilissima*	NMEMC
2009.08.07	Bohai Sea	120.9833	37.4500	*Heterosigma akashiwo*	NMEMC
2009.08.18-22	East China Sea	118.1833	24.5356	*Skeletonema costatum*	NMEMC
2009.08.19-21	East China Sea	122.6917	30.7667	*Thalassiosira rotula*	NMEMC

2009.08.20	Bohai Sea	121.5614	37.5025	*Gymnodinium sanguineum*	NMEMC
2009.08.26	South China Sea	115.3506	22.7625	*Phaeocystis globosa*	NMEMC
2009.10.22	South China Sea	113.7285	22.3948	*Thalassiosira nordenskioeldii*	NMEMC
2009.10.26	South China Sea	113.8968	22.4581	*Gyrodinium instriatum*	NMEMC
2009.10.27	South China Sea	113.6333	22.3333	*Cochlodinium polykrikoides*	NMEMC
2009.11.01-13	South China Sea	115.3503	22.7631	*Phaeocystis globosa*	NMEMC
2009.11.09	South China Sea	113.5744	22.2864	Phaeocystis globosa	NMEMC

Note: “-”indicated algal species was not reported by the National Marine Environmental Monitoring Center (NMEMC).

Table S3. Recorded information on the total number and total area of HABs in the four areas of China.

Start year	South China Sea		East China Sea		Yellow Sea		Bohai Sea		Source
	Total number	Total area (km^2)	Total number	Total area (km^2)	Total number	Total area (km^2)	Total number	Total area (km^2)	
1995	1	10	13	70	1	95	2	250	China Marine Disaster Bulletin
1996	2	30	9	1100	1	45	0	0	China Marine Disaster Bulletin
1997	1	10	4	30	1	10	3	24	China Marine Disaster Bulletin
1998	9	100	4	10	2	160	7	3800	China Marine Disaster Bulletin
1999	6	430	1	50	4	20	5	8700	China Marine Disaster Bulletin
2000	6	50	11	7800	4	800	7	2000	China Marine Disaster Bulletin
2001	15	300	34	8400	8	4600	20	2800	China Marine Disaster Bulletin
2002	11	540	51	9000	4	550	13	100	China Marine Disaster Bulletin
2003	16	830	86	13500	5	90	12	130	China Marine Disaster Bulletin
2004	17	1330	56	17500	13	700	12	7180	China Marine Disaster Bulletin
2005	9	630	51	19000	13	1600	9	5000	China Marine Disaster Bulletin
2006	17	1270	63	15170	2	420	11	2980	China Marine Disaster Bulletin
2007	10	496	60	9787	5	655	7	672	China Marine Disaster Bulletin
2008	8	60	47	12070	12	1578	1	30	China Marine Disaster Bulletin
2009	8	391	43	6554	13	1878	4	5279	China Marine Disaster Bulletin
2010	14	223	39	6374	9	735	7	3560	China Marine Disaster Bulletin
2011	11	190	23	1427	8	4242	13	217	China Marine Disaster Bulletin
2012	16	741	38	2028	11	1333	8	3869	China Marine Disaster Bulletin
2013	6	167	25	1573	2	450	13	1880	China Marine Disaster Bulletin
2014	16	684	27	2509	2	19	11	4078	China Marine Disaster Bulletin
2015	12	141	15	1098	1	48	7	1522	China Marine Disaster Bulletin
2016	17	968	37	5714	4	62	10	740	China Marine Disaster Bulletin
2017	13	1048	40	2189	3	100	12	342	China Marine Disaster Bulletin
